# Fear effect on the mobility of individuals in a spatially heterogeneous environment: a delayed diffusive SPIR epidemic model

**DOI:** 10.1038/s41598-025-16280-2

**Published:** 2025-08-24

**Authors:** Ghilmana Sarmad, Salih Djilali, Soufiane Bentout, Abdessamad Tridane

**Affiliations:** 1 Department of Mathematical Sciences, United Arab Emirates University, P.O. Box 15551, Al Ain, UAE; 2 Department of Mathematics, Faculty of Exact Sciences and Informatics, Hassiba Benbouali University, 02000 Chlef, Algeria; 3 Department of Mathematics and Informatics, University Ain Temouchent, Belhadj Bouchaib, BP 284 RP, 46000 Ain Témouchent, Algeria

**Keywords:** Delayed epidemic model, Diffusion, Nonlocal diffusion, protection, Fear effect, Infectious diseases, Psychology and behaviour

## Abstract

As the fear of infection is a crucial factor in the progress of the disease in the population. We aim, in this study, to investigate a susceptible-protected-infected-recovered (SPIR) epidemic model with mixed diffusion modeled by local and nonlocal diffusions. These types of diffusion are used to model the fear effect of being infected by the population. The model is shown to be well-posed; the solution exists, is positive, and is unique. The variational expression is obtained to determine threshold role of $\mathfrak {R}_0$, also known as the basic reproduction number. Indeed, for $\mathfrak {R}_0<1$, we show that the epidemic will extinct, corresponding to the global asymptotic stability of the infection-free equilibrium state. However, when $\mathfrak {R}_0>1$, the existence of the infection equilibrium state and the uniform persistence of the solution have been proved. The Lyapunov function have been used to show the global asymptotic stability of the infection equilibrium state. Moreover, we compared the obtained results with the classical *SIR* epidemic model for determining the required protection function for stopping the disease, which can be obtained by reducing $\mathfrak {R}_0$ below one.

## Introduction

In the worst-case scenarios, any infectious that can cause severe sickness or even death can be associated with fear in society. Like any other emotion, this fear of infection is contagious and can spread rapidly. As a change of behavior due to the pandemic crisis, fear can lead individuals to “homestay,” on the individual level or on the household level^1^, to avoid contact with the population and getting the infection. This consequently led to “agoraphobia”^2^ in certain people, as was the case during the recent Covid-19 pandemic, when many governments were implementing large-scale restrictions on the population mobility as a measure to protect the population from the spread of the disease. The natural question is: To what extent is “homestay”, as a protection measure, effective in reducing the impact of the pandemic?

Scarcely, no models have incorporated the impact of fear, even though it is easily spread. A mathematical and computational model that integrated both fear dynamics and contagion disease was presented by Epstein et al.^3^. Valle et al.^4^ studied a model that discusses the impact of fear-based homestay as behavior change on the expansion of pandemic influenza. But the question still remains if it is enough to stop the progress of the pandemic.

In investigating and understanding the spread of infectious diseases, mathematical and computational models play a significant role. The objective of these investigations is to anticipate the consequences of some specific public health interventions such as vaccination^5^, treatment^6^, education^7^, media effect^8^, lockdown^9^ when applied to contain the spread of disease. These studies have been very helpful in discovering new public health safety measures and determining the most efficient measure that would dampen the disease under consideration. Interestingly, the non-infected class rigorously practices and respects the new health interventions as they become cautious about getting infected.

The spread of disease among people can be reduced through educational campaigns and some controlling mechanisms^10^. One such control measure is the homestay, which we will refer to in our paper as “protection.” An observation indicates that the susceptible class is the one that is scared of getting infected the most. Moreover, adapting the mode of protection varies from person to person within the non-infected class, as some would have preferred vaccination while others would have opted for isolation or maybe the combination of these.

It is to highlight that the global dynamics of a delayed epidemic model is governed by its basic reproduction number $\mathfrak {R}_0$, and the goal of carrying out protection-related studies is to find the protection effort required to reduce $\mathfrak {R}_0$ below one. Finding the most efficient protective system for different populations has always been challenging. Mezouaghi et al.^11^ used a similar approach to find the required minimal protection rate $b_{2\min }$ to stop an epidemic. In^12^, the protection measure’s effect on the spread of infection was studied using an age-structured model in which the obtained basic reproduction number represented the equilibrium states, which were also globally stable.

Any outbreak of a disease is highly influenced by its spatial dispersion. Notably, a high spatial diffusion can dampen a disease. Based on a similar idea, many scholars and researchers have investigated the spatiotemporal effect on infectious disease^13–16^. These models have been further categorized to include local diffusion and nonlocal dispersion to analyze the spread of the disease. Han and Lei propose one such model^14^ where they have infused local diffusion in a diffusive SIR epidemic model in a heterogeneous environment. Moreover, the former category is represented by a Laplacian operator ($\Delta$), which many scholars also use to predict the process of the disease spread among different populations such as^17,18^ and the references therein.

It is to be noted that to date, all proposed mathematical models either use the local dispersion^15,16,19–21^ or the nonlocal dispersion^13,14,17,18,22^. Our paper introduces a noble approach to building a spatiotemporal model that infuses both local and nonlocal dispersion to investigate the effect of fear on the spread of the disease. In our model, the extremely careful movements of the non-infected populace (the susceptible and the protected category) due to the fear of getting infected across the studied area $\Gamma$ (assumed as a bounded set in $\mathbb {R}$) is considered by the local-diffusion operator (Laplacian operator or random diffusion). By definition, the random diffusion represents that the movements are done in the neighborhood of the original point *x*. This behavior is modeled by using the S-equation and P-equation of a delayed epidemic model. Here, it is worth mentioning that most movements are used for extreme needs, such as running errands. Moreover, we can’t emphasize less that it is hard to study fear without the spatial movements of the individuals as they are highly influenced by it. On the other hand, after becoming infected, a person with agoraphobia may experience increased mobility, as they may need to visit hospitals and other locations, leading to a significant rise in their movement compared to their initial (susceptible) status. This type of behavioral change is also observed with some sexually transmitted diseases, where individuals may initially overemphasize precautions, potentially contributing to agoraphobia. Media coverage can further amplify agoraphobia within the population. Numerous diseases, such as COVID-19, influenza, chickenpox, hepatitis A, and Ebola, have shown similar influences on mobility.

On the other hand, after being infected the individuals will lose their precaution, and become more free to move, and the phobia from being infected will be gone. In this case, the movement of the ones that been infected, that includes infected and recovered persons, are more often and the radius of the movement in no longer in the neighborhood of the original point. Therefore, the random diffusion no longer models the spatial movements of this category. To model this, we will use different dispersal operator, that is nonlocal diffusion^23–27^, that defined as

$$\mathbb {H}[z](x):=\displaystyle \int _\Gamma J(x-y)(z(y)-z(x))dy,$$

with *J* is the probability distribution of leaping from position *y* to location *x*. consequently, each individual sinks to location *x* at the rate $\int _\Omega J(x-y)\phi (y)dy$ and abandons his place at that rate. The support of the function *J* can be considered to cover more area than the neighbor of the location *x*, for the purpose of modelling losing caution obtain after being infected. For more reading on the properties, and the basic theory of the nonlocal diffusion operator, we refer the excellent references^23–25^, and for the recent achievements on the nonlocal diffusion problems, we refer^26,28–34^. Notably, Mezouaghi et al.^11^ is a special case of what follows next. Therefore, we propose the following delay epidemic model, which is given after infusing all assumptions mentioned above:

1 $$\begin{aligned} \left\{ \begin{array}{rlll} \frac{\partial }{\partial t}S(t,x)& =& \delta _S \Delta S+ b_3(x) -b_1(x) S(t,x)I(t,x)-(b_4(x)+b_2(x)) S(t,x)+\varepsilon b_2 e^{-b_4\tau } S(t-\tau ,x),\\ \frac{\partial }{\partial t}P(t,x)& =& \delta _P \Delta P(t,x)+ b_2(x) S(t,x)-b_4(x) P(t,x)-\varepsilon b_2(x) e^{-b_4(x)\tau } S(t-\tau ,x),\\ \frac{\partial }{\partial t}I(t,x) & =& \delta _{I} \mathbb {H}[I](t,x)+ b_1(x) S(t,x)I(t,x)-(b_4(x)+b_5(x)+b_6(x)) I(t,x),\\ \frac{\partial }{\partial t} R(t,x)& =& \delta _{R} \mathbb {H}[R](t,x)+ b_5(x) I(t,x)-b_4(x) R(t,x),\end{array} \right. \end{aligned}$$

with $t>0$ and $x \in \Gamma$. The associated initial and boundary conditions

2 $$\begin{aligned} \left\{ \begin{array}{rlll} \frac{\partial S(t,x)}{\partial \textbf{n}}& =& 0,~~\frac{\partial P(t,x)}{\partial \textbf{n}}=0,& x \in \partial \Gamma \\ S(s,x)& =& S_0(s,x),~~P(0,x)=P_{0}(x),~~I(0,x)=I_{0}(x),~~R(0,x)=R_{0}(x),& s\in [-\tau ,0[,~x \in \bar{\Gamma }, \end{array} \right. \end{aligned}$$

where the susceptible populace *S*(*t*, *x*), the protected class of individuals *P*(*t*, *x*), the infected population *I*(*t*, *x*), and the recovered class of persons *R*(*t*, *x*) are defined at time *t* and position *x*. $\delta _S$, $\delta _P$, $\delta _I$, $\delta _R$ are diffusion coefficients. At position *x*, $b_3$ gives the birth coefficient. $b_4$ and $b_5$ represent (for every unit of time) the natural death and recovery rates, respectively. $b_6$ is the death rate due to infection at location *x*. $b_2$ is the protection rate at location *x*, and $\epsilon$ is the proportion of the protected population that finished the protection period and became susceptible again. The term $\varepsilon b_2 e^{-b_4\tau }S(t-\tau ,x)$ represents the population entering into the protected class at time $t-\tau$, completing the duration of protection $\tau$, leaving the isolation and then becoming susceptible again at time *t* and location *x*. This type of modeling is motivated by the approach^35^. Moreover, the system considers the Neumann boundary conditions (1), for more information on the difference of Neumann and Dirichlet boundary conditions for nonlocal diffusion, we refer^23^. Also, the model coefficients take on the following assumptions:

**(A1)**: $\delta _S,\delta _P,\delta _I,\delta _R\ge 0$, with $\delta _S,\delta _I$ are not both zero.
**(A2)**: On $\bar{\Gamma }$, the strictly positive $b_4$ and $b_3$ are also Lipschitz continuous .
**(A3)**: On $\bar{\Gamma }$, $b_6$, $b_2$ $b_5$, and $b_1$ are nonnegative and continuous.
**(A4)**: On $\mathbb {R}$, the Lipschitz function *J*(*x*) satisfies
  $$1=\int _{\mathbb {R} }J(x)dx, ~ J(x) \text{ is } \text{ strictly } \text{ positive } \text{ on } \bar{\Gamma }\text{, } \text{ and } \int _\Gamma J(x-y)dy\not \equiv 1\text{, } J(x)=J(-x) \text{ on } \mathbb {R} \text{. }$$

Determining the global dynamics of the model (1) is the true motivation of our paper. Moreover, we compare the dynamics of (1) with the classical SIR epidemic model to characterize the influence of the protection on reducing the epidemic. We will proceed by taking into consideration the effect of the protection force $b_2$ on the containment of the epidemic and by assuming that $\mathfrak {R}_0 ^{SIR}>1$ (the basic reproduction number corresponding to the SIR model). The focus of this paper is to search for the optimal protection force $b_2$ so that the basic reproduction number $\mathfrak {R}_0$ of (1) becomes less than one, which leads eventually to the containment of the epidemic. This protection force $b_2$ not only influences the temporal behavior of the solution but also has a strong relationship with the fear among the individuals.

To achieve this goal, the manuscript is organized as follows. In section “Basic results”, we provide some basic results to show that our system is well-posed, i.e., its solution exists, is unique, and is positive. Further, we have shown that the model admits a unique infection-free equilibrium state (IFEs). We concluded the section by calculating the corresponding $\mathfrak {R}_0$. In section “IFEs”, the Lyapunov function has been used to prove that IFEs when $\mathfrak {R}_0<1$, is globally stable. Section “IEs” is provided with the proof of the existence of the infection equilibrium state (will be denoted by IEs), the solution, and its uniform persistence when $\mathfrak {R}_0>1$. Moreover, we have shown that the IEs is globally asymptotically stable when one of the diffusion coefficients $\delta _i,~~ i=1,2$ is a zero. In section “Relationship between local and corresponding nonlocal diffusion”, we determined the relationship between local and nonlocal diffusion and discussed how nonlocal diffusion generalizes local diffusion. In section “The situation of the epidemic influenced by the protection force”, we have presented our findings about how protection is helpful in stopping the epidemic by calculating the required minimal protection force. Our mathematical results have been supported numerically in section “Numerical investigation of the results”.

## Basic results

To facilitate our the notations, $f(x)= f,~x\in \bar{\Gamma }$, where $f= b_1, b_2,b_3,b_4, b_5,b_6$. We further consider $f^-=\min \{f(x),~~x\in \bar{\Gamma }\}$, with $f \in C(\bar{\Gamma })$ .

Let

$$\mathbb {Y}= \mathbb {C} ([-\tau ,0]\times \bar{\Gamma },\mathbb {R})\times \mathbb {C} (\bar{\Gamma },\mathbb {R})\times \mathbb {C}(\bar{\Gamma },\mathbb {R})\times \mathbb {C}(\bar{\Gamma },\mathbb {R}),$$

be a Banach space equipped with the norm

$$\Vert \psi \Vert =\sup \limits _{s\in [-\tau ,0),~~t\in \bar{\Gamma }} \sqrt{|\psi _1(s,x)|^2+|\psi _2(x)|^2+|\psi _3(x)|^2+|\psi _4(x)|^2},\qquad \psi =(\psi _1,\psi _2,\psi _3,\psi _4)\in \mathbb {Y},$$

and the positive cone in $\mathbb {Y}$ is

$$\mathbb {Y}_+= \mathbb {C} ([-\tau ,0]\times \bar{\Gamma },\mathbb {R}^+)\times \mathbb {C} (\bar{\Gamma },\mathbb {R}^+)\times \mathbb {C}(\bar{\Gamma },\mathbb {R}^+)\times \mathbb {C}(\bar{\Gamma },\mathbb {R}^+).$$

Defining the linear operator

$$A \tilde{U}=\left( \begin{array}{c} A_1 S\\ A_2 P\\ A_3 I \\ A_4 R \end{array}\right) =\left( \begin{array}{c} \delta _S \Delta S(t,\cdot )-(b_4+b_2) S(t,\cdot )\\ \delta _P \Delta P(t,\cdot )-b_4 P(t,\cdot )\\ \delta _{I} \mathbb {H}[I](t,\cdot )- \delta _{I} I(t,\cdot )-(b_4+b_5+b_6) I(t,\cdot )\\ \delta _{R} \mathbb {H}[R](t,\cdot )-b_4 R(t,\cdot ) \end{array}\right) ,$$

and

$$F\tilde{U}=\left( \begin{array}{c} b_3 -b_1 S(t,\cdot )I(t,\cdot )+\varepsilon b_2 e^{-b_4\tau } S(t-\tau ,\cdot )\\ b_2 S(t,\cdot )+\varepsilon b_2 e^{-b_4\tau } S(t-\tau ,\cdot )\\ b_1 S(t,\cdot )I(t,\cdot )\\ b_5 I(t,\cdot ) \end{array}\right) .$$

Hence, (1) can be formulated as:

3 $$\begin{aligned} \frac{d\tilde{U}}{dt}=F[\tilde{U}](t)+A[\tilde{U}](t),\quad \tilde{U}(t) \in \mathbb {Y}_+. \end{aligned}$$

We got the following the result:

### Lemma 1

*A strictly contractive and uniformly continuous semi-group* $\{ e ^{At}\}_{t\ge 0}$ *is generated by* *A*. *Moreover, for all* $t\ge 0$, $e ^{At}\mathbb {Y}_+$ *is contained in* $\mathbb {Y}_+$

### Proof

We decompose the operator *A* as $A=A_0+A_1$, with

$$A_0 \tilde{U}=\left( \begin{array}{c} -(b_4+b_2) S(t,\cdot )\\ -b_4 P(t,\cdot )\\ -(\delta _{I} \tilde{J}(x)+b_4 +b_5 +\delta ) I(t,\cdot )\\ -( \delta _{R}\tilde{J}(x)+b_4 )R(t,\cdot ) \end{array}\right) ,\quad A_1 \tilde{U}=\left( \begin{array}{c} \delta _S \Delta S(t,\cdot )\\ \delta _P \Delta P(t,\cdot )\\ \delta _{I} \displaystyle \int _\Gamma J(\cdot -y)I(t,y)dy\\ \delta _{R} \displaystyle \int _\Gamma J(\cdot -y)R(t,y)dy \end{array}\right) ,$$

with $\tilde{J}(x)=\int _\Gamma J(x-y)dy$, and $U\in \mathbb {Y}$. Hence $\{ e ^{At}\}_{t\ge 0}$, a strictly contractive and uniformly continuous semi-group is generated by $A_0$ on $\mathbb {Y}$. Using presumption **(A4)**, we deduce that $A_1$ is bounded. Thus we concluded that *A* produces a positive continuous semi-group $\{ e ^{At}\}_{t\ge 0}$ with the help of^36, Theorem 1.2]^ and ^37, Corollary VI 1.11]^ resulting in the desired result. $\square$

The result about the solution’s existence is given next:

### Theorem 1

*Suppose that* $\tilde{U}_0=\left( \psi _1,\psi _2,\psi _3,\psi _4\right) \in \mathbb {Y}$. *Hence, there exists* $t_0>0$ *that ensures the solution for* (1), *denoted by* $U(.,U_0)\in \mathbb {C}((0,t_0),\mathbb {Y})\cap \mathbb {C}^1([0,t_0],\mathbb {Y})$, *exists and is unique and verifies that either* $t_0=\infty$ *or* $\limsup _{t\rightarrow t_0 ^-}\Vert \tilde{U}(t,\tilde{U}_0)\Vert =\infty$ *holds. Furthermore, if* $\tilde{U}_0\in \mathbb {Y}_+$, *then* $\tilde{U}(t,\tilde{U}_0)\in \mathbb {Y}_0$ *for* $t\in (0,t_0)$.

### Proof

Expressed below is the solution of (1):

4 $$\begin{aligned} \tilde{U}(t)=\tilde{U}_0 e^{At}+ \displaystyle \int _0 ^t e^{A(t-s)}F(\tilde{U}(s))ds. \end{aligned}$$

Clearly, *F* is a $C^1$ functional. Then,^38, Proposition 4.16]^ guarantees that unique solution denoted by $\tilde{U}(.,\tilde{U}_0)\in \mathbb {C}([0,t_0],\mathbb {Y})\cap \mathbb {C}^1([0,t_0],\mathbb {Y})$ for (1) exists and verifies $t_0=\infty$ or $\limsup _{t\rightarrow t_0 ^-}\Vert \tilde{U}(t,\tilde{U}_0)\Vert =\infty .$ Now, for $t\in (0,t_0)$ and $x\in \bar{\Gamma }$ we have $S(t,x)>0$ . Assuming that there exists $x_0\in \Gamma$ and $\tilde{t}\in (0,t_0)$ verifying $S(\tilde{t},x_0)=0$, and $S(t,x_0)>0$ for $t<\tilde{t}$. Then, we can define

$$t_1=\inf \{t\in (0,t_0):S(t,x_0)=0\}.$$

Clearly, $t_1\in (0,t_0),$ with $S(t_1,x_0)=0$ such that $\dfrac{\partial S (t_1,x_0)}{\partial t}\le 0.$ The first eq. of (1) yields

$$\dfrac{\partial S (t_1,x_0)}{\partial t}=b_3(x_0)+\epsilon b_2(x_0) e^{-b_4(x_0)\tau } S(t_1-\tau ,x_0)>0,$$

contradicting our supposition. Hence, for $t\in (0,t_0)$, $S(t,x)>0$ and $x\in \bar{\Gamma }$.

For sufficiently large $h>0$, we let $F(\tilde{U}(t,.))+h \tilde{U}(t,.)= F_h(\tilde{U})$. Evidently, for $B\left( 0,\dfrac{1}{h}\right)$ which represents an open ball centered at 0 with radius $\dfrac{1}{h}$ in $\mathbb {Y}$, we can have a positive $F_h(\tilde{U})$ for $\tilde{U}\in B\left( 0,\dfrac{1}{h}\right) \cap \mathbb {C}((0,t_0),\mathbb {Y})$. Hence, (1) can be written as:

$$\dfrac{d\tilde{U}(t,\cdot )}{dt}=F_h(\tilde{U}(t,\cdot ))+A_h \tilde{U}(t,\cdot ).$$

Then, the solution is

$$\tilde{U}(t,x)= \tilde{U}_0(x) e^{A_h t}+\displaystyle \int _0 ^t e^{A_h (t-s)}F_h(\tilde{U}(s,x))ds.$$

As a result, for $t\in (0,t_0)$, $\tilde{U}(t,U_0)\in \mathbb {Y}_+$ , for $\tilde{U}_0\in \mathbb {Y}_+.$ $\square$

Next, we let $\{\tilde{\Theta }(t)\}_{t\ge 0}:R^+\times \mathbb {Y}_+\rightarrow \mathbb {Y}_+$ be the semiflow associated to the system (1), that is defined as

$$\tilde{\Theta }(t,U)=(S(t,\cdot ),P(t,\cdot ),I(t,\cdot ),R(t,\cdot )),~~t\ge 0,~~\tilde{U}\in \mathbb {Y}_+.$$

We use the following theorem to check the well-posedness of the solution:

### Theorem 2

$\tilde{U}(t,\tilde{U}_0)$ *with* $\tilde{U}_0\in \mathbb {Y}_+$ *for* (1) *is the unique solution. Hence,the unique global solution is* $\tilde{U}(t,\tilde{U}_0):[0,+\infty )\rightarrow \mathbb {Y}_+$. *Moreover,* (1) *generates a bounded dissipative semiflow* $\{\tilde{\Theta }(t)\}_{t\ge 0}$.

### Proof

Denote $$N(t)=\int _\Gamma S(t,x)+P(t,x)+I(t,x)+R(t,x)dx$$, then

$$\begin{array}{lll} \frac{dN(t)}{dt}& =& \int _\Gamma \frac{\partial S(t,x)}{\partial t}+\frac{\partial P(t,x)}{\partial t}+\frac{\partial I(t,x)}{\partial t}+\frac{\partial R(t,x)}{\partial t}dx,\\ & =& \int _\Gamma b_3-b_4 (S(t,x)+P(t,x)+I(t,x)+R(t,x)) -b_6 (x) I(t,x) dx,\\ & \le & \int _\Gamma b_3-b_4 (S(t,x)+P(t,x)+I(t,x)+R(t,x)) dx,\\ & \le & \int _\Gamma b_3 dx -b_4^- N(t), \end{array}$$

with $b_4^-=\min _{x\in \bar{\Gamma }} \{b_4\}$. We infer that if $N(0)\le \frac{\int _\Gamma b_3 dx}{b_4^-}$ then for $t\in [0,t_0)$, $N(t)\le \frac{\int _\Gamma b_3 dx}{b_4^-}$ using the variation constant method. Next, we have $\tilde{S}(t,x)\ge S(t,x)$ for all $t>0$ and $x\in \Gamma$ by the standard comparison principle, where $\tilde{S}(t,x)$ satisfies

$$\left\{ \begin{array}{ll} \frac{\partial }{\partial t}\tilde{S}(t,x)=\delta _S \Delta \tilde{S}(t,x)+ b_3-(b_4+b_2) \tilde{S}(t,x)+\epsilon b_2 e^{-b_4\tau } \tilde{S}(t-\tau ,x),& t>0,~~x \in \Gamma ,\\ \tilde{S}(s,x)=S_0(s,x), & s\in [-\tau ,0[,~~x\in \bar{\Gamma }, \end{array}\right.$$

with the Neumann boundary conditions. It is easy to show that $\tilde{S}(t,x)\rightarrow S^0(x)$ as $t\rightarrow \infty$ (the detailed proof will be done later in step 1 section “Uniform persistence”). Therefore, we have $\limsup _{t\rightarrow \infty }\Vert S\Vert \le K_S,~~t\ge 0$ for some positive constant $K_S$ that depends on the initial data. The boundedness of $P-$equation can be deduced from *S* being bounded. Moreover, based on the fact that $\int _\Gamma Idx$ is bounded i.e. $\exists$ a constant $\tilde{K_I}>0$ such that $\int _\Gamma Idx\le \tilde{K_I},~\forall$  $t\in [0,t_0]$, then we have

$$\frac{\partial }{\partial t}I(t,x) \le \delta _{I} \tilde{K_I}- \delta _{I} I(t,x)+ b_1 K_sI(t,x)-(b_4+b_5+b_6) I(t,x),~~t>0,~~x \in \Gamma .$$

A simple calculation implies that a constant *M* (that depends on the site of $\max \{b_1 K_sI(t,x)-(b_4+b_5+b_6), ~~x\in \bar{\Gamma }\}$ exists such that $\Vert I(t,x)\Vert \le K_I e^{Mt}$, with $K_I$ depending only on the initial data. Therefore, from Theorem 1 we conclude that $t_0=\infty$ has a globally well-posed solution. The previous conclusions imply that the semiflow $\{\tilde{\Theta }(t)\}_{t\ge 0}$ is globally defined and is bounded dissipative by the boundedness result. $\square$

The first and third equations in (1) do not contain *P* and *R*. So, the following delayed spatiotemporal model is obtained by reducing (1):

5 $$\begin{aligned} \left\{ \begin{array}{rlll} \frac{\partial }{\partial t}S(t,x)& =& \delta _S \Delta S(t,x)+ b_3 -b_1 S(t,x)I(t,x)-(b_4+b_2) S(t,x)\\ & & +\epsilon b_2 e^{-b_4\tau } S(t-\tau ,x),& t>0,~~x \in \Gamma ,\\ \frac{\partial }{\partial t}I(t,x) & =& \delta _{I} \mathbb {H}[I](t,x)+ b_1 S(t,x)I(t,x)\\ & & -(b_4+b_5+b_6) I(t,x),& t>0,~~x \in \Gamma ,\\ \frac{\partial S(t,x)}{\partial \textbf{n}}& =& 0,& t\ge 0,~~x \in \partial \Gamma , \\ S(s,x)& =& S_0(s,x),~~I(0,x)=I_{0}(x),& s\in [-\tau ,0[~~x \in \bar{\Gamma }. \end{array} \right. \end{aligned}$$

with $$t>0$$ and $$x \in \Gamma$$.

### Remark 1

By analyzing the asymptotic behavior of the reduced system represented by equation (5), we can deduce remaining equations through the application of the comparison principle. This involves demonstrating that the supremum and infimum are equal.

Now we define a Banach space

$$\mathbb {X}= \mathbb {C} ([-\tau ,0]\times \bar{\Gamma },\mathbb {R})\times \mathbb {C}(\bar{\Gamma },\mathbb {R}),$$

With the following norm:

$$\Vert \psi \Vert =\sup \limits _{s\in [-\tau ,0),~~x\in \bar{\Gamma }} \sqrt{|\psi _1(s,x)|^2+|\psi _2(x)|^2},\qquad \psi =(\psi _1,\psi _2)\in \mathbb {X},$$

and $\mathbb {X}_+= \mathbb {C}([-\tau ,0]\times \bar{\Gamma },\mathbb {R}^+)\times \mathbb {C}(\bar{\Gamma },\mathbb {R}^+)$ be the positive cone in $\mathbb {X}$.

Define the semiflow that corresponds to the system (5) as $\{\Theta (t)\}_{t\ge 0}:R^+\times \mathbb {X}_+\rightarrow \mathbb {X}_+$, which is formulated as follows:

$$\Theta (t,U)=(S(t,\cdot ),I(t,\cdot )),~~t\ge 0,~~U\in \mathbb {X}_+.$$

$\{\Theta (t)\}_{t\ge 0}$ is also bounded dissipative since $\{\tilde{\Theta }(t)\}_{t\ge 0}$ is bounded dissipative semiflow from Theorem 2.

### Existence of IFEs

Letting $E^0=(S^0,0)\in \mathbb {C}(\bar{\Gamma },\mathbb {R}^+)\times \mathbb {C}(\bar{\Gamma },\mathbb {R}^+)$ is the IFEs for (5), where $S^0$ is the solution of the problem:

6 $$\begin{aligned} \left\{ \begin{array}{lll} 0=\delta _S \Delta S^0(x)+ b_3-\left( b_4+b_2-\varepsilon b_2 e^{-b_4\tau }\right) S^0(x),& x \in \Gamma ,\\ \frac{\partial S^0(x)}{\partial \textbf{n}}=0,& x \in \partial \Gamma . \end{array} \right. \end{aligned}$$

The next lemma guarantees the existence and the uniqueness of $S^0$ in $\mathbb {C}(\bar{\Gamma },\mathbb {R}^+).$

#### Lemma 2

*The IFEs,* $E^0=(S^0,0)\in \mathbb {X}_+$ *is unique for the system* (5).

#### Proof

The existence of IFEs can be shown by determining the zeros of the following function:

$$H(\psi )(x):=\delta _S \Delta \psi + b_3-\left( b_4+b_2-\varepsilon b_2 e^{-b_4\tau }\right) \psi ,~~x \in \Gamma .$$

In order to determine the root $S^0(x)$ of $H(S^0(x))=0$, a lower and upper solution denoted by $\underline{S}^0(x)$ and $\overline{S}^0(x)$ respectively, will be formulated verifying:

$$H(\underline{S}^0(x))\ge 0,~~H(\overline{S}^0(x))\le 0,~~and~~ \overline{S}^0(x)\ge \underline{S}^0(x)\ge 0,~~x\in \Gamma .$$

Since $H(0)=b_3>0$, $\exists$ $\mathfrak {m}>0$ sufficiently small implying that $H(\mathfrak {m})>0$. We then put $\underline{S}^0(x)=\mathfrak {m}$. Similarly, we consider $\mathfrak {M}>0$ is a positive constant sufficiently large that verifies $\mathfrak {M}>\mathfrak {m}$. Moreover, we put $\overline{S}^0(x)=\mathfrak {M}$. Thus,

$$H(\mathfrak {M})=b_3-\left( b_4+b_2-\varepsilon b_2 e^{-b_4\tau }\right) \mathfrak {M}.$$

Since $\lim _{\mathfrak {M}\rightarrow \infty }H(\mathfrak {M})=-\infty$, then $\mathfrak {M}$ exists and verifies $H(\mathfrak {M})<0$. Therefore, at least one strictly positive solution is guaranteed for (6). Next, we show the uniqueness of $S^0(x)$. We suppose that (6) has another solution $\tilde{S}_0(x)\in \mathbb {C}(\bar{\Gamma })\backslash \{0\}$ such that $\tilde{S}_0\not \equiv S^0$, which satisfies the problem.

7 $$\begin{aligned} \left\{ \begin{array}{lll} 0=\delta _S \Delta \tilde{S}^0(x)+ b_3-\left( b_4+b_2-\varepsilon b_2 e^{-b_4\tau }\right) \tilde{S}^0(x),& x \in \Gamma ,\\ \frac{\partial S^0(x)}{\partial \textbf{n}}=0,& x \in \partial \Gamma . \end{array}\right. \end{aligned}$$

From (6), we have $b_3=-\delta _S \Delta S+\left( b_4+b_2-\varepsilon b_2 e^{-b_4\tau }\right) S^0(x)$, and by replacing this result into (7) and letting $\bar{S}^0(x)=\tilde{S}^0(x)-S^0(x)$, we get

8 $$\begin{aligned} \left\{ \begin{array}{lll} 0=\delta _S \Delta \bar{S}^0(x)-\left( b_4+b_2-\varepsilon b_2 e^{-b_4\tau }\right) \bar{S}^0(x),& x \in \Gamma ,\\ \frac{\partial \bar{S}^0(x)}{\partial \textbf{n}}=0,& x \in \partial \Gamma . \end{array}\right. \end{aligned}$$

Clearly, the unique solution is given by $\bar{S}^0(x)=0$. Hence, $\tilde{S}^0(x)=S^0(x)$, $x\in \bar{\Gamma }$. Then $E^0$ is unique. The proof is completed $\square$

### Existence of a Global compact attractor

#### Lemma 3

*If* $S_0(s,x)\le S^0(x),~x\in \bar{\Gamma }$ *and* ${s\in {[-\tau ,0[}}$, *then we have* $S(t,x)\le S^0(x)$ *for all* $t\ge 0,~x\in \bar{\Gamma }$.

#### Proof

From (6), we have

$$b_3=-\delta _S \Delta S^0 (x) +(b_4+b_2-\epsilon b_2 e^{-b_4\tau })S^0 (x),~~x \in \Gamma .$$

Let $\tilde{S}(t,x)=S^0(x)-S(t,x)$,  $t\ge 0$ and $x\in \bar{\Gamma }$. Then,

$$\begin{array}{lll} \frac{\partial }{\partial t} \tilde{S}(t,x)& =& -\frac{\partial }{\partial t} S(t,x),\\ & =& -\delta _S \Delta S(t,x)- b_3 +b_1 S(t,x)I(t,x)+(b_4+b_2) S(t,x)+\epsilon b_2 e^{-b_4\tau } S(t-\tau ,x),\\ & =& \delta _S \Delta \tilde{S}(t,x)+b_1 S(t,x)I(t,x)+(b_4+b_2) \tilde{S}(t,x)+\epsilon b_2 e^{-b_4\tau } \tilde{S}(t-\tau ,x),\\ & \ge & \delta _S \Delta \tilde{S}(t,x)+(b_4+b_2 )\tilde{S}(t,x)+\epsilon b_2 e^{-b_4\tau } \tilde{S}(t-\tau ,x), ~~t\in [0,t_0),~~x\in \Gamma . \end{array}$$

We let $\bar{S}(t,x)$ be the solution of the problem

9 $$\begin{aligned} \left\{ \begin{array}{rllll} \frac{\partial }{\partial t}\bar{S}(t,x)& =& \delta _S \Delta \bar{S}(t,x)-(b_4+b_2) \bar{S}(t,x)+\epsilon b_2 e^{-b_4\tau } \bar{S}(t-\tau ,x),~~t>0,~& x \in \Gamma ,\\ \frac{\partial \bar{S}(t,x)}{\partial \textbf{n}}& =& 0,& x \in \partial \Gamma ,~t\ge 0,\\ \bar{S}(s,x)& =& S_0(s,x)-S^0(x)\ge 0,& s\in [-\tau ,0[,~~x \in \bar{\Gamma }. \end{array} \right. \end{aligned}$$

Using similar reasoning as in Theorem 1, we get $\bar{S}(t,x)\ge 0$ for all $t\ge 0$ and $x\in \bar{\Gamma }$. Standard comparison principle implies that $\tilde{S}(t,x)\ge \bar{S}(t,x)\ge 0$, $t\ge 0$ and $x\in \bar{\Gamma }$. Therefore, $S^0(x)\ge S(t,x)$, $x\in \bar{\Gamma }$. We conclude the proof. $\square$

Let

10 $$\begin{aligned} \Sigma =\left\{ U\in \mathbb {X}: S(.,x)\le S^0(x),~~x\in \bar{\Gamma },~~\displaystyle \int _\Gamma S(t,x)dx+\displaystyle \int _\Gamma I(t,x)dx\le \frac{\int _\Gamma b_3 dx}{b_4 ^-} \right\} , \end{aligned}$$

and

$$\Sigma _0 =\left\{ U\in \Sigma : I(.,x)>0 ~~for ~~ x \in \bar{\Gamma }\right\} .$$

Note that the positively invariant set of (5) is $\Sigma$. Now, with the help of the next lemma, we will prove that $\Sigma _0$ is also a positively invariant set.

#### Lemma 4

*We suppose that* (5) *has a unique solution* *U*(*t*, *x*) *with* $U_0\in \Sigma _0$ *then for all* $t>0$, $x\in \bar{\Gamma }, ~I(t,x)>0$.

#### Proof

We consider the problem

11 $$\begin{aligned} \left\{ \begin{array}{llll} \frac{\partial }{\partial t}\bar{I}(t,x) =\delta _{I} \mathbb {H}[\bar{I}](t,x)-(b_4+b_5+b_6) \bar{I}(t,x),~~t>0,& x \in \Gamma ,\\ \bar{I}(0,x)=I_{0}(x),& x \in \bar{\Gamma }. \end{array} \right. \end{aligned}$$

Clearly, $I(t,x)\ge \bar{I}(t,x)$, where $\bar{I}(t,x)$ is the solution of (11). Now, we only need to prove that $\bar{I}(t,x)>0$ for all $t>0$ and $x\in \bar{\Gamma }$. The inspiration for the proof comes from proof of^39, Proposition 2.2]^. We begin by defining $P:\mathbb {C}(\bar{\Gamma })\rightarrow \mathbb {C}(\bar{\Gamma })$ as

$$PI(t,x)=\delta _{I} \displaystyle \int _\Gamma J(x-y)\bar{I}(t,y)dy,~~x \in \bar{\Gamma }.$$

Note that *P* is continuous and generates a uniformly continuous semigroup $\{ e^{Pt}\}_{t\ge 0}$ which is also positive on $\mathbb {C}(\bar{\Gamma })$, where we have $e^{Pt}=\Sigma _{n=0} ^{\infty }\frac{t^n P^n}{n!}$ for $t\ge 0$ . Moreover,

$$e^{Pt}(I_0(x))=\Sigma _{n=0} ^{\infty }\frac{t^n P^n(I_0(x))}{n!},~~t>0.$$

We have $P^{n+1}(I_0(x))=\displaystyle \int _\Gamma J(x-y)P^n I_0(y)dy,$ for $x \in \bar{\Gamma }$ and $n\ge 1$ utilizing the fact that $\bar{I}(t,x)\ne 0$. We deduce the existence of $n_0$ with the iterative technique, verifying $P^{n+1}(I(0,x))>0,~~x \in \bar{\Gamma }$ and $n\ge n_0$. Thus, $P^{n}(I_0(x))>0,~~x \in \bar{\Gamma }$. Therefore,

$$\begin{aligned} \left\{ \begin{array}{rlll} \bar{I}(t,x) & =& e^{- (\delta _{I}+b_4+b_5+b_6) t } e^{Pt}(I_0(x))+\displaystyle \int _0 ^t e^{- (\delta _{I}+b_4+b_5+b_6)(t-\sigma ) } e^{P(t-\sigma )}\bar{I}(\sigma ,x)d\sigma ,\\ & \ge & e^{- (\delta _{I}+b_4+b_5+b_6)t } e^{Pt}(I_0(x))>0, \end{array} \right. \end{aligned}$$

for $$t>0$$, $$x\in \bar{\Gamma }$$. $$\square$$

Now, we look into the asymptotic smoothness of the semigroup (check^40, Definition 2.25]^). We will now prove that $\{\Theta (t)\}_{t\ge 0}$ (the semiflow) is compact. We first present the definition as given below:

#### Definition 1

^41^ Let $g: X\rightarrow X$ be a continuous map. *g* is $\varkappa -condensing$ if the bounded sets are mapped by *g* to the bounded sets and $\varkappa (g(A))<\varkappa (A)(\varkappa (g(A))<l\varkappa (A))$ with $0\le l<1$ for every closed bounded nonempty set $A\in X$, with $\varkappa (A)$ is Kuratowski measure that is $\varkappa (A):=\inf \{r:\text {the finite cover of } A \text { has diameter~ <r }\}$. Therefore, $\varkappa (A)=0$ if and only if *A* is precompact. Hence, the $\varkappa -condensing$ maps exhibit asymptotic smoothness (check ^42, Lemma 2.3.5]^).

The following theorem gives the asymptotic smoothness of $\{\Theta (t)\}_{t\ge 0}$ (the semiflow) by:

#### Theorem 3

*Assume that either* $\delta _S$ *or* $\delta _I$ *is a zero, then* $\{\Theta (t)\}_{t\ge 0}$ *(the semiflow)* *is* $\varkappa -condensing$. *Furthhermore, there is a global compact attractor in* $\mathbb {X}_+$ *for semiflow* $\{\Theta (t)\}_{t\ge 0}$.

#### Proof

We consider $U_0=(S_0,I_0)\in \mathbb {X}_+$. The semiflow $\{\Theta (t)\}_{t\ge 0}$ associated to (5) is $\Theta (t)=(S(t,\cdot ),I(t,\cdot ))$.

- If $\delta _I=0$: In this case, the semiflow $\{\Theta (t)\}_{t\ge 0}$ becomes non-compact. Clearly, the second equation of the system (5) can be expressed as
  $$I(t,\cdot )= e^{-(b_4+b_5+b_6) t}I(0,\cdot )+b_1 \displaystyle \int _0 ^t e^{-(b_4+b_5+b_6) (t-s)}I(s,\cdot )S(s,\cdot )ds.$$
  With initial data $U_0\in C(\Omega )$, we denote by $S(t,x;U_0)$ (resp. $I(t,x;U_0)$), the solution of (5). By applying Arezela–Ascoli Theorem as^43, Lemma 2.5]^, we deduce that for any bounded $B\subset \textbf{X}_+$ and $t>0$, the set
  $$\mathcal {S}:=\left\{ \displaystyle \int _0 ^t e^{-(b_4+b_5+b_6) (t-s)}b_1 I(s,\cdot ;U_0)S(s,\cdot ;U_0)ds:U_0\in B \right\} ,$$
  is precompact in $C(\bar{\Omega })$, and hence $\varkappa (\mathcal {S})=0$. Next, we decompose our semiflow $\Theta (t)$ as $\Theta (t)=F_1(t)+F_2(t),$ $t\ge 0$, where
  $$F_1(t)U_0=\left( S(t,\cdot ;U_0),\displaystyle \int _0 ^t e^{-(b_4+b_5+b_6) (t-s)}b_1 I(s,\cdot ;U_0)S(s,\cdot ;U_0)ds \right) ,~~t\ge 0,$$
  and
  $$F_2(t)U_0=\left( 0, e^{-(b_4+b_5+b_6) t}I(0,\cdot ) \right) ,~~t\ge 0.$$
  Let us suppose a bounded set $B\subseteq \textbf{X}_+$. By Theorem 2, for any $t>0$ the set $\{F_1(s)B,s\in [0,t]\}$ is bounded. For any $t>0$, $F_1(t)B$ is precompact since $\mathcal {S}$ is precompact in $C(\bar{\Omega })$. Thus $\varkappa (F_1(t)B)=0,~~t>0$. Moreover,
  $$\varkappa (F_2(t)B)\le \Vert e^{-(b_4^-+b_5^-+b_6 ^-) t}\Vert \varkappa (B)\le e^{-(b_4^-+b_5^-+b_6 ^-) t}\varkappa (B),~~t\ge 0.$$
  Therefore,
  $$\varkappa (\Theta (t)B)\le \varkappa (F_1(t)B)+\varkappa (F_2(t)B)\le \kappa (t)\varkappa (B).$$
  where $\kappa (t)= e^{-(b_4^-+b_5^-+b_6 ^-) t}$, which means that $\Theta (t)$ is a $\varkappa -contraction$.
- If $\delta _S=0$: Here, the semiflow $\{\Theta (t)\}_{t\ge 0}$ is also non-compact. Clearly the first equation of the system (5) can be expressed as
  $$S(t,\cdot )= e^{-(b_4+b_2) t}S_0+\displaystyle \int _0 ^t e^{-(b_4+b_2) (t-s)}\Bigg [b_3-b_1 I(s,\cdot )S(s,\cdot )+ \varepsilon b_2 e^{-b_4\tau } S(t-\tau ,\cdot ) \bigg ]ds.$$
  Similarly, the Arezela–Ascoli Theorem implies that for any bounded $B\subset \textbf{X}_+$ and $t>0$, the set given by
  $$\mathcal {S}_1:=\left\{ \displaystyle \int _0 ^t e^{-(b_4+b_2) (t-s)}\Bigg [b_3-b_1 I(s,\cdot ;U_0)S(s,\cdot ;U_0)+ \varepsilon b_2 e^{-b_4\tau } S(t-\tau ,\cdot ;U_0) \bigg ]ds:U_0\in B \right\} ,$$
  is precompact in $C(\bar{\Omega })$, and hence $\varkappa (\mathcal {S}_1)=0$. Again, we decompose our semiflow $\Theta (t)$ as $\Theta (t)=F_1(t)+F_2(t),$ $t\ge 0$, where
  $$F_1(t)U_0=\left( \displaystyle \int _0 ^t e^{-(b_4+b_2) (t-s)}\Bigg [b_3-b_1 I(s,\cdot ;U_0)S(s,\cdot ;U_0)+ \varepsilon b_2 e^{-b_4\tau } S(t-\tau ,\cdot ;U_0) \bigg ]ds,I(t,\cdot ;U_0) \right) ,$$
  and
  $$F_2(t)U_0=\left( e^{-(b_4+b_2) t}S(0,\cdot ), 0 \right) ,$$
  for $t\ge 0.$ We suppose another bounded set $B_1\subseteq \textbf{X}_+$. Therefore, for any positive *t*, the set $\{F_1(s)B_1, s\in [0,t]\}$ is bounded. Moreover, for any $t>0$, $F_1(t)B_1$ is precompact. Thus $\varkappa (F_1(t)B_1)=0,~~t>0$. Furthermore,
  $$\varkappa (F_2(t)B_1)\le \Vert e^{-(b_4^-+b_2^-) t}\Vert \varkappa (B_1)\le e^{-(b_4^-+b_2^-) t}\varkappa (B_1),~~t\ge 0.$$
  Therefore,
  $$\varkappa (\Theta (t)B_1)\le \varkappa (F_1(t)B_1)+\varkappa (F_2(t)B_1)\le \kappa _1(t)\varkappa (B_1).$$
  with $\kappa _1(t)= e^{-(b_4^-+b_2^-) t}$. Thus, the first part of the proof is finished.

We will now proceed with the final segment of our proof. From Theorem 1–2, we infer that the global solution of (5) exists and is unique. Since $\Sigma$ defined by (10) is a positively invariant set, the semiflow $\Theta (t),~t\ge 0$ is point dissipative. If neither $\delta _S$ nor $\delta _I$ is a zero then the semiflow is compact, and consequently, the semigroup $T(t),~~t\ge 0$ is completely continuous, and by^42, Corolary 3.2]^ it is asymptotically smooth. However, if either $\delta _S$ or $\delta _I$ is a zero, then the semiflow $T(t),~~t\ge 0$ is not compact, but it is $\varkappa -$condensing, by applying^42, Lemma 3.2.5]^, we deduce the asymptotic smoothness of $T(t),~~t\ge 0$. Therefore,^42, Theorem 3.4.6]^ implies a global compact attractor. $\square$

### Basic reproduction number

In (5), equation number two is linearized at IFEs, resulting in the governing equation of the infection component

12 $$\begin{aligned} \frac{\partial }{\partial t}I(t,x) = \delta _{I} \mathbb {H}[I](t,x)+ b_1 S^0(x)I(t,x)-(b_4+b_5+b_6) I(t,x), \end{aligned}$$

for $t>0$ and $x \in \bar{\Gamma }$. Considering $\mathfrak {F}$ (the linear operator) defined on $\mathbb {C} (\bar{\Gamma })$ by

$$\mathfrak {F}\psi (x):= b_1 S^0(x)\psi (x),\qquad \psi \in \mathbb {C} (\bar{\Gamma }),$$

and the linear operator $A_I$ defined by

$$A_I \phi (x)=\delta _{I} \mathbb {H}[\phi ](x)+ b_1 S^0(x)\phi (x)-(b_4+b_5+b_6) \phi (x),~~x\in \Gamma .$$

We can write (12) in the abstract form in $\mathbb {C} (\bar{\Gamma })$ with the help of the operator $A_{I}$ as follows:

$$\frac{dI(t)}{dt}=A_{I}I(t)+\mathfrak {F}I(t),\qquad t>0.$$

Observe $s(A_{I})<0$ represents that $A_{I}$ is resolvent-positive,. Thus,

$$(-A_{I})^{-1}\psi (x)=\int _0 ^\infty T_{I}(t)\psi (x)dt, \qquad \psi \in \mathbb {C} (\bar{\Gamma }).$$

We present the next generation operator $\chi :=\mathfrak {F}(-A_{I})^{-1}$ (see^44^) as follows:

$$\chi \psi (x)=b_1 S^0(x)\int _0 ^\infty T_{I}(t)\psi (x)dt,\qquad \psi \in \mathbb {C} (\bar{\Gamma }).$$

$\mathfrak {R}_0$ representing the basic reproduction number is given by

$$\mathfrak {R}_0:=r(\chi ).$$

Where spectral radius *r* belongs to the operator. Inspired by^45^, the eigenvalue problem associated to the problem (12) is

13 $$\begin{aligned} \lambda \psi (x)=\delta _{I} \mathbb {H}[\psi ](x)+ b_1 S^0(x)\psi (x)-(b_4+b_5+b_6) \psi (x),~~x\in \Gamma . \end{aligned}$$

Based on the fact that $b_1 S^0(x)-(b_4+b_5+b_6)$ is Lipschitz on $\bar{\Gamma }$ corresponding to the eigenfunction $v_0(x)>0$, we get the principal eigenvalue of (13) denoted by $\mu _0$ (see^46^) where

$$\mu _0=-\inf _{\psi \in L^2 (\Gamma )}\left\{ \frac{1}{2}\delta _I \displaystyle \int _\Gamma \displaystyle \int _\Gamma J(x-y)(v(y)-v(x))^2dydx- \displaystyle \int _\Gamma \left[ b_1 S^0(x)-(\delta _{I} +b_4+b_5+b_6) \right] v^2(x)dx\right\} .$$

Based on the definition of $\mu _0$, we have $\mu _0=s(A_I+\mathfrak {F})$. Since that $A_I$ is resolvent-positive and $s(A_I)<0$, we have $sign \{s(A_I+\mathfrak {F})\}=sign\{r(\mathfrak {F}(-A_I ^{-1}))-1\}$. Consequently, $sign\{\mu _0\}=sign\{\mathfrak {R}_0-1\}$.

By applying^46, Theorem 1.4]^, we obtain that the following expression

14 $$\begin{aligned} \mathfrak {R}_0=\sup _{\psi \in L^2 (\Gamma )}\left\{ \frac{ \displaystyle \int _\Gamma b_1 S^0(x)(\psi (x))^2dx}{\delta _I \displaystyle \int _\Gamma \displaystyle \int _\Gamma J(x-y)(\psi (y)-\psi (x))^2dydx+\displaystyle \int _\Gamma (b_4+b_5+b_6)(\psi (x))^2dx}\right\} . \end{aligned}$$

defines the the variational expression of the basic reproduction number.

#### Remark 2

Clearly, $R_0$ goes to 0 as $\delta _I$ tends to $+\infty$. Therefore, we deduce that diffusion can contain the epidemic.

## IFEs

To our interest, we will now show the global stability of the IFEs that is $E^0(S^0(x),0),~x\in \bar{\Gamma }$ in the following section. The next theorem presents the results we have obtained.

### Theorem 4

*IFEs are globally asymptotically stable for* $\mathfrak {R}_0<1$ *in* $\Sigma$.

### Proof

We choose a Lyapunov function given below to begin with

$$L_1(t):=\displaystyle \int _\Gamma v_0(x) I(t,x)dx,$$

with $v_0(x)$ corresponding to $mu_0$ is a strictly positive eigenfunction. Clearly, $L_1\ge 0$ and $L_1=0$ if and only if $I\equiv 0$. Therefore, we perform the derivation of $L_1$ in the following manner:

$$\begin{array}{lll} \frac{d}{dt}L_1(t)& =& \displaystyle \int _\Gamma v_0(x) \frac{\partial }{\partial t}I(t,x)dx,\\ & =& \displaystyle \int _\Gamma v_0(x) \left[ \delta _{I} \displaystyle \int _\Gamma J(x-y)I(t,y)dy+ b_1 S(t,x)I(t,x)-(\delta _{I}\tilde{J}+b_4+b_5+b_6) I(t,x)\right] dx. \end{array}$$

with $\tilde{J}=\int _\Gamma J(x-y)dy$ Note that

$$\begin{array}{lll} \displaystyle \int _\Gamma \delta _{I} v_0(x) \displaystyle \int _\Gamma J(x-y)I(t,y)dydx& =& \displaystyle \int _\Gamma \delta _{I} I(t,y) \displaystyle \int _\Gamma J(x-y)v_0(x)dx dy,\\ & =& \displaystyle \int _\Gamma \delta _{I} I(t,x) \displaystyle \int _\Gamma J(x-y)v_0(y)dydx ,\\ & =& \displaystyle \int _\Gamma \delta _{I} I(t,x) \left[ \mu _0 v_0(x)- b_1 S^0(x)v_0(x)+(\delta _{I}\tilde{J}+b_4+b_5+b_6) v_0(x) \right] dx. \end{array}$$

Then, $$\frac{d}{dt}L_1(t)$$ becomes

$$\begin{array}{lll} \frac{d}{dt}L_1(t)& =& \displaystyle \int _\Gamma v_0(t) \left[ \mu _0 I(t,x)+ b_1 I(t,x)(S-S^0(x))\right] dx,\\ & \le & \displaystyle \int _\Gamma v_0(t) \mu _0 I(t,x)dx,\\ & \le & 0. \end{array}$$

For $\mathfrak {R}_0<1$, we have $\mu _0<0$, then $\frac{d}{dt}L_1(t)\le 0$, and based on the fact that $v_0(x)>0$, $x\in \bar{\Gamma }$, we get if $I(t,x)=0$ then $\frac{d}{dt}L_1(t)=0$ and vice versa. Therefore, $I\rightarrow 0$ as $t\rightarrow \infty$. Hence, for all $\vartheta >0$ (we choose it to be sufficiently small) there is $t_1>0$ (sufficiently large), such that for all $t>t_1$ we have $I(t,x)<\vartheta$ in $\Gamma$. Therefore, for $t>t_1$, we have $S(t,x)\le \hat{S}(t,x)$, where $\hat{S}$ is the unique solution of the following problem

15 $$\begin{aligned} \left\{ \begin{array}{rlll} \frac{\partial }{\partial t}\hat{S}& =& \delta _S \Delta \hat{S}+ b_3 -(b_1\vartheta + b_4+b_2) \hat{S}+\epsilon b_2 e^{-b_4\tau } \hat{S}_\tau ,& t>t_1,~~x \in \Gamma ,\\ \frac{\partial \hat{S}}{\partial \textbf{n}}& =& 0,& t\ge t_1,~~x \in \partial \Gamma , \end{array} \right. \end{aligned}$$

with initial condition belongs to $\Sigma$. We claim that $\hat{S}\rightarrow \hat{S}^0$, with $\hat{S}^0$ is the unique positive solution of the elliptic problem

16 $$\begin{aligned} \left\{ \begin{array}{lll} 0=\delta _S \Delta S^0(x)+ b_3-\left( b_1 \vartheta + b_4+b_2-\varepsilon b_2 e^{-b_4\tau }\right) S^0(x),& x \in \Gamma ,\\ \frac{\partial S^0(x)}{\partial \textbf{n}}=0,& x \in \partial \Gamma , \end{array} \right. \end{aligned}$$

then, by (16), we get

$$b_3=-\delta _S \Delta S^0 (x) +(b_1 \vartheta + b_4+b_2-\epsilon b_2 e^{-b_4\tau })S^0 (x),~~x \in \Gamma .$$

Suppose $\hat{S}^0(x)-\hat{S}= \tilde{S}(t,x)$, $t\ge t_2>t_1,~x\in \bar{\Gamma }$, with $t_2$ is sufficiently large. Then,

$$\begin{array}{lll} \frac{\partial }{\partial t} \tilde{S}(t,x)& =& -\frac{\partial }{\partial t} S,\\ & =& -\delta _S \Delta S- b_3 +(b_4+b_2) S+\epsilon b_2 e^{-b_4\tau } S_\tau ,\\ & =& \delta _S \Delta \tilde{S}(t,x)+(b_1 \vartheta +b_4+b_2) \tilde{S}(t,x)+\epsilon b_2 e^{-b_4\tau } \tilde{S}(t-\tau ,x),\\ & =& \delta _S \Delta \tilde{S}(t,x)+(b_1 \vartheta +b_4+b_2) \tilde{S}(t,x)+\epsilon b_2 e^{-b_4\tau } \tilde{S}(t-\tau ,x), ~t\ge 0,~x\in \Gamma . \end{array}$$

Therefore, the problem outlined below

17 $$\begin{aligned} \left\{ \begin{array}{rlll} \frac{\partial }{\partial t}\tilde{S}(t,x)& =& \delta _S \Delta \tilde{S}(t,x)-(b_1 \vartheta +b_4+b_2) \tilde{S}(t,x)+\epsilon b_2 e^{-b_4\tau }\tilde{S}(t-\tau ,x),& t>0,~~x \in \Gamma ,\\ \frac{\partial \tilde{S}(t,x)}{\partial \textbf{n}}& =& 0,& t\ge 0,~~x \in \partial \Gamma , \\ \end{array} \right. \end{aligned}$$

has a solution $\tilde{S}(t,x)$. We are now going to prove that as $t\rightarrow \infty$, $\tilde{S}(t,x)$ goes to 0. Let us consider the eigenvalue problem given below:

18 $$\begin{aligned} \left\{ \begin{array}{lll} \lambda _0 \psi (x)=\delta _S \Delta \psi (x)-\left( b_1 \vartheta +b_4+b_2-\varepsilon b_2 e^{-b_4\tau }\right) \psi (x),& x \in \Gamma ,\\ \frac{\partial \psi (x)}{\partial \textbf{n}}=0,& x \in \partial \Gamma , \end{array}\right. \end{aligned}$$

has principal eigenvalue $\lambda _0=0$, and $\psi (x)$ is the corresponding eigenfunction which is strictly positive in $\Gamma$. Now, taking the eigenvalue problem corresponding to (17) into consideration:

19 $$\begin{aligned} \left\{ \begin{array}{lll} \lambda ^* \phi (x)=\delta _S \Delta \phi (x)-(b_4+b_2+\varepsilon b_2 e^{-b_4\tau } e^{-\lambda ^*\tau } )\phi (x),& x \in \Gamma ,\\ \frac{\partial \phi (x)}{\partial \textbf{n}}=0,& x \in \partial \Gamma , \end{array}\right. \end{aligned}$$

with $\phi (x)$ is also positive on $\Gamma$. Now we multiply (18) by $\phi$ and (19) by $\psi$ and add them, then integrate by parts on $\Gamma$ which gives

$$\lambda ^*\displaystyle \int _\Gamma \phi (x)\psi (x)dx=\displaystyle \int _\Gamma \varepsilon b_2 e^{-b_4\tau }\phi (x)\psi (x)\left( e^{-\lambda ^*\tau }-1 \right) dx.$$

We assume that $\lambda ^*>0$. We get that $e^{-\lambda ^*\tau }-1<0$, which leads to a contradiction as it implies opposite signs on the right-hand side (negative) and the left-hand side (positive). Therefore, $\lambda ^*<0$. By supposing that $\tilde{S}(t,x)= e^{\lambda ^*(t-t_0)}\phi (x)$, with $t_0$ sufficiently large, we get $\tilde{S}(t,x)$ goes to 0 as $t\rightarrow \infty$. Similarly, we can show that for $t>t_1$ we have $I(t,x)>-\vartheta$ in $\Gamma$ (with $\vartheta$ is chosen very small as necessary). $S(t,x)\ge \breve{S}(t,x)$, where $\breve{S}$ is the unique solution of the following problem

20 $$\begin{aligned} \left\{ \begin{array}{rlll} \frac{\partial }{\partial t}\hat{S}& =& \delta _S \Delta \hat{S}+ b_3 -(-b_1\vartheta + b_4+b_2) \hat{S}+\epsilon b_2 e^{-b_4\tau } \hat{S}_\tau ,& t>t_1,~~x \in \Gamma ,\\ \frac{\partial \hat{S}}{\partial \textbf{n}}& =& 0,& t\ge t_1,~~x \in \partial \Gamma , \end{array} \right. \end{aligned}$$

with initial condition belongs to $\Sigma$. We obtain that $\breve{S}\rightarrow \breve{S}^0$, with $\breve{S}^0$ solves

21 $$\begin{aligned} \left\{ \begin{array}{lll} 0=\delta _S \Delta S^0(x)+ b_3-\left( -b_1 \vartheta + b_4+b_2-\varepsilon b_2 e^{-b_4\tau }\right) S^0(x),& x \in \Gamma ,\\ \frac{\partial S^0(x)}{\partial \textbf{n}}=0,& x \in \partial \Gamma , \end{array} \right. \end{aligned}$$

Then we obtain that

$$0<\breve{S}^0\le \lim _{t\rightarrow \infty }S(t,x)\le \hat{S}^0.$$

Because $\vartheta$ deduces that the solution is continuous, we let $\vartheta \rightarrow 0$, which implies that $S(t,x)\rightarrow S^0$. Then, in $\Sigma$, $E_0$ is globally stable, and that concludes the proof. $\square$

## IEs

This section analyzes for $\mathfrak {R}_0>1$, the behavior of the solution of (5). For this aim, we put the following subsections.

To facilitate our the notations, we are using $S_X=S(t,x)$ and $S_{\tau ,x}=S(t-\tau ,x)$, and $I_x=I(t,x)$.

### Uniform persistence

#### Definition 2

Let $\zeta$ be a sufficiently small positive number. If this $\zeta$ exists such that it verifies for any $U_0\in \mathbb {X}_+$, $U(t,U_0)$ satisfies

$$\liminf _{t\rightarrow \infty }I_x>\zeta ,~~x\in \bar{\Gamma },$$

then we have a uniformly persistent system given by (5).

We give the definition of the spaces as follows:

$$\partial \Sigma _0 =\left\{ U\in \Sigma : I(.,x)=0 ~~for ~~ x \in \bar{\Gamma }\right\} ,$$

$$\Sigma _\partial =\left\{ U_0\in \partial \Sigma _0: \Theta (t,U_0)\in \partial \Sigma _0 ~~for ~~ t\ge 0 \right\} .$$

To show this result, we need to verify all axioms of^40, Theorem 3]^ through the following steps:

**Step 1:** Global stability of $E^0$ for semiflow $\{\Theta (t)\}_{t\ge 0}$ is restricted to $\partial \Sigma _0$.

Clearly, $\partial \Sigma _0$ and $\Sigma _\partial$ are positively invariant sets (see Lemma 4). Next, we show that the semiflow $\{\Theta (t)\}_{t\ge 0}$ restricted to $\partial \Sigma _0$ has globally asymptotically stable IFEs for initial condition belonging to $\Sigma _\partial$. In this case, *S* is governed by the equation

22 $$\begin{aligned} \left\{ \begin{array}{rlll} \frac{\partial }{\partial t}S_x& =& \delta _S \Delta S_x+ b_3 -(b_4+b_2) S_x+\varepsilon b_2 e^{-b_4\tau } S_{\tau ,x},& t>0,~~x \in \Gamma ,\\ \frac{\partial S_x}{\partial \textbf{n}}& =& 0,& t\ge 0,~~x \in \partial \Gamma . \end{array} \right. \end{aligned}$$

We take the following Lyapunov function into consideration:

$$\tilde{L}(t)=\tilde{L}_1(t)+\tilde{L}_2(t),$$

with

$$\tilde{L}_1(t)=\displaystyle \int _\Gamma S^0 (x)h\left( \frac{S_x}{S^0(x)}\right) dx,$$

and

$$\tilde{L}_2(t)=\displaystyle \int _\Gamma \varepsilon b_2 e^{-b_4\tau }(S^0(x))^2 \displaystyle \int _0 ^\tau h\left( \frac{S_{s,x}}{S^0(x)}\right) dsdx.$$

Here, *h* represents a Volterra function defined as $h(x)=x-1-\ln x$. The problem (22) along with the derivative of $\tilde{L}_1(t)$ is

$$\begin{array}{rlll} \frac{d }{d t}\tilde{L}_1(t)& =& \displaystyle \int _\Gamma S^0(x)\left( 1-\frac{S^0(x)}{S_x}\right) \left( \delta _S \Delta S_x+b_3-(b_4+b_2) S_x+\varepsilon b_2 e^{-b_4\tau }S_{\tau ,x}\right) dx,\\ & =& \displaystyle \int _\Gamma S^0(x)\left\{ \left( 1-\frac{S^0(x)}{S_x}\right) \left( \delta _S \Delta S_x+b_3\right) +\left( 1-\frac{S_x}{S^0(x)}\right) \delta _S \Delta S^0(x)\right. \\ & & \left. +\varepsilon b_2 e^{-b_4\tau } S^0(x) \left( 1-\frac{S_x}{S^0(x)}+\frac{S_{\tau ,x}}{S^0(x)}-\frac{S_{\tau ,x}}{S_x}\right) +\left( 1-\frac{S_x}{S^0(x)}\right) b_3\right\} dx,\\ & =& \displaystyle \int _\Gamma S^0(x)\left\{ \left( 2-\frac{S^0(x)}{S_x}-\frac{S_x}{S^0(x)}\right) b_3+\delta _S \Delta S_x\left( 1-\frac{S^0(x)}{S_x}\right) +\left( 1-\frac{S_x}{S^0(x)}\right) \delta _S \Delta S^0(x)\right. \\ & & \left. +\varepsilon b_2 e^{-b_4\tau } S^0(x) \left( 1-\frac{S_x}{S^0(x)}+\frac{S_{\tau ,x}}{S^0(x)}-\frac{S_{\tau ,x}}{S_x}\right) \right\} dx. \end{array}$$

Now, we calculate $\frac{d }{d t}\tilde{L}_2(t)$ which is given as

$$\begin{array}{lll} \frac{d }{d t}\tilde{L}_2(t)& =& \frac{d }{d t}\displaystyle \int _\Gamma \varepsilon b_2 e^{-b_4\tau }(S^0(x))^2 \displaystyle \int _0 ^\tau h\left( \frac{S_{s,x}}{S^0(x)}\right) dsdx,\\ & =& \displaystyle \int _\Gamma \varepsilon b_2 e^{-b_4\tau }(S^0(x))^2 \frac{\partial }{\partial t}\displaystyle \int _0 ^\tau h\left( \frac{S_{s,x}}{S^0(x)}\right) dsdx . \end{array}$$

Note that

$$\begin{array}{lll} \frac{\partial }{\partial t}\displaystyle \int _0 ^\tau h\left( \frac{S_{s,x}}{S^0(x)}\right) ds & =& \displaystyle \int _0 ^\tau \frac{\partial }{\partial t} h\left( \frac{S_{s,x}}{S^0(x)}\right) ds,\\ & =& -\displaystyle \int _0 ^\tau \frac{\partial }{\partial s} h\left( \frac{S_{s,x}}{S^0(x)}\right) ds,\\ & =& -\left. h\left( \frac{S_{s,x}}{S^0(x)}\right) \right| _0 ^\tau ,\\ & =& - \frac{S_{\tau ,x}}{S^0(x)}+\frac{S_x}{S^0(x)}+\ln \left( \frac{S_{\tau ,x}}{S_x}\right) , \end{array}$$

thus, we get

$$\begin{array}{lll} \frac{d }{d t}\tilde{L}_2(t)= & \displaystyle \int _\Gamma \varepsilon b_2 e^{-b_4\tau }(S^0(x))^2 \left[ - \frac{S_{\tau ,x}}{S^0(x)}+\frac{S_x}{S^0(x)}+\ln \left( \frac{S_{\tau ,x}}{S_x}\right) \right] dsdx. \end{array}$$

Hence,

$$\begin{array}{lll} \frac{d }{d t}\tilde{L}(t)& =& \displaystyle \int _\Gamma S^0(x)\left\{ \left( 2-\frac{S^0(x)}{S_x}-\frac{S_x}{S^0(x)}\right) b_3+\delta _S \Delta S_x\left( 1-\frac{S^0(x)}{S_x}\right) +\left( 1-\frac{S_x}{S^0(x)}\right) \delta _S \Delta S^0(x)\right. \\ & & \left. -\varepsilon b_2 e^{-b_4\tau } S^0(x) h\left( \frac{S_{\tau ,x}}{S_x}\right) \right\} dx. \end{array}$$

Now, it remains to show that

$$M(t)=\delta _S\displaystyle \int _\Gamma S^0(x)\left\{ \delta _S \Delta S_x\left( 1-\frac{S^0(x)}{S_x}\right) +\left( 1-\frac{S_x}{S^0(x)}\right) \delta _S \Delta S^0(x)\right\} dx \le 0.$$

Using Green’s first identity and Neuman boundary conditions, we get

$$\begin{array}{lll} M(t)& =& \delta _S\displaystyle \int _\Gamma S^0(x)\left\{ \Delta S_x\left( 1-\frac{S^0(x)}{S_x}\right) +\left( 1-\frac{S_x}{S^0(x)}\right) \Delta S^0(x)\right\} dx,\\ & =& \delta _S\left\{ \displaystyle \int _{\partial \Gamma } S^0(x)\left( 1-\frac{S^0(x)}{S_x}\right) \nabla S_x. \textbf{n}~dS-\displaystyle \int _\Gamma \nabla \left( S^0(x)\left( 1-\frac{S^0(x)}{S_x} \right) \right) .\nabla S_x dx\right. \\ & & \left. +\displaystyle \int _{\partial \Gamma } S^0(x)\left( 1-\frac{S_x}{S^0(x)}\right) \nabla S^0(x).\textbf{n}~dS-\displaystyle \int _\Gamma \nabla \left( S^0(x)\left( 1-\frac{S_x}{S^0(x)} \right) \right) .\nabla S^0(x)dx\right\} ,\\ & =& -\delta _S\displaystyle \int _\Gamma \left( \frac{d S^0(x)}{d x}-2\frac{S^0(x)}{S_x}\frac{d S^0}{dx}+\frac{(S^0(x))^2}{(S_x)^2} \frac{\partial S_x}{\partial x} \right) \frac{\partial S_x}{\partial x}dx \\ & & -\delta _S\displaystyle \int _\Gamma \left( \frac{d S^0(x)}{d x}-\frac{S_x}{S^0(x)}\right) \frac{d S^0(x)}{d x}dx,\\ & =& -\delta _S\displaystyle \int _\Gamma \left( \frac{d S^0(x)}{dx}-\frac{S^0(x)}{S_x}\frac{\partial S_x}{\partial x} \right) ^2dx,\\ & \le & 0. \end{array}$$

Therefore, $\frac{d }{d t}\tilde{L}(t)$ is non-positive. The equality holds *iff*  $S_{\tau ,x}=S_x=S^0(x)$, $x\in \bar{\Gamma }$. Thus, we obtain a globally stable $E^0$ in $\partial \Sigma _0$ for $\mathfrak {R}_0>1$.

**Step 2:** Uniform weak repeller.

Here, we will show that $\Sigma$ has a uniform weak repeller, the IFEs. Hence, we need to show that a sufficiently small positive constant denoted by $l>0$ exists in a way that the semiflow $\{\Theta \}_{t\ge 0}$ will satisfy

$$\limsup _{t\rightarrow \infty }\Vert \Theta (t,U_0)-E^0\Vert \ge l.$$

This result will be shown by contradiction. We assume that $\limsup _{t\rightarrow \infty }\Vert \Theta (t,U_0)-E^0\Vert \le l$. Then $\exists t_1$ so that the solution $U(t,U_0)$ verifies

$$S^0(x)-l\le S_x\le S^0(x)+l,~~0<I_x\le l,~~t\ge t_1, ~~x\in \bar{\Gamma }.$$

Since that $\mathfrak {R}_0>1$, $l_1$ denoting a sufficiently small constant, exists and satisfies $I(t_1,x)>l_1v_0(x)$ with $v_0(x)\in X_+$. Note, $(\mu _0,v_0)$ is the eigenvalue and corresponding eigenfunction which verifies the following problem:

23 $$\begin{aligned} \mu _0 v_0(x)=\delta _{I} \mathbb {H}[v_0](x)+ b_1(S^0(x)-l_0)v_0(x)-(b_4+b_5+b_6) v_0(x),~~x\in \Gamma . \end{aligned}$$

Hence, for the problem given below, we consider $\tilde{I}(t,x)$ as a solution.

24 $$\begin{aligned} \left\{ \begin{array}{rlll} \frac{\partial }{\partial t}\tilde{I}(t,x) & =& \delta _{I} \mathbb {H}[\tilde{I}](t,x) + b_1(S^0(x)-l_0)\tilde{I}(t,x)-(b_4+b_5+b_6) \tilde{I}(t,x),\\ \tilde{I}(t_1,x)& =& l_1v_0(x), \end{array} \right. \end{aligned}$$

with $$t>t_1,~~x \in \Gamma ,$$ Therefore,

$$I_x \ge \tilde{I}(t,x)= e^{\mu _0(t-t_1)}l_2 v_0(x),~t\ge t_1,~x \in \bar{\Gamma }.$$

Since $\mu _0$ is positive for $\mathfrak {R}_0>1$, as $t\rightarrow \infty$ we get $\Vert I_x\Vert \rightarrow \infty$ contradicting our supposition.

**Step 3:** Uniform persistence.

Taking the assumption that the orbit $\gamma ^+(U_0):=\bigcup _{t\ge 0}\{\Theta (t)\}$ has $\omega (U_0)$ as the $\omega -limit$ set. Moreover, $\Theta (t,\Sigma _0)\subseteq \Sigma _0$ and $\Theta (t,\partial \Sigma _0)\subseteq \partial \Sigma _0$ are obtained. Therefore, $\Sigma _\partial =\partial \Sigma _0$, and $\omega (U_0)=E^0$ for every $U_0\in \partial \Sigma _0$. Hence, $E^0$ is isolated in $\mathbb {X}$. Since $\omega (U_0)=E^0$ for all $U_0\in \partial \Sigma _0$, then there is no cycle in $\partial \Sigma _0$ from $E_0$ to $E_0$.

From the results of step 2, we get $W^s(E^0)\bigcap \Sigma _0=\emptyset$, with $W^s(E^0)=\{U_0\in X:~~\lim _{t\rightarrow \infty }\Theta (t,U_0)=E^0\}$. Therefore, the system (5) is uniformly persistent for $\mathbb {R}_0>1$ as all axioms mentioned in^40, Theorem 3]^ holds. The proof is completed.

### Existence of IEs

#### Theorem 5

*The system admits at least one IEs whenever* $\mathfrak {R}_0>1$.

#### Proof

We have obtained the point dissipativeness of semiflow $\Theta (r,U_0)$ from theorem 2. Also, Theorem 3 guarantees that there exists an attractor that is globally compact and attracts all points from $\Sigma _0$. Since $\Sigma _0$ is convex and $\{\Theta (r)\}_{r\ge 0}$ (the semiflow) is $\varkappa -$condensing (see Theorem 3), $\{\Theta (r)\}_{r\ge 0}$ has a steady state $E^*=(S^*,I^*)\in \Sigma _0$ by^47, Theorem 4.7]^. Moreover, the persistence result shows that (5) has a strictly positive solution, i.e., the IEs, which verifies the following mix elliptic problem:

25 $$\begin{aligned} \left\{ \begin{array}{rlll} 0& =& \delta _S \Delta S^*(x)+ b_3 -b_1 S^*(x)I^*(x)-(b_4+b_2-\epsilon b_2 e^{-b_4\tau }) S^*(x),& x \in \Gamma ,\\ 0 & =& \delta _{I}\mathbb {H}[I^*](x)+ b_1 S^*(x)I^*(x)-(b_4+b_5+b_6) I^*(x),& x \in \Gamma .\\ \frac{\partial S^*(x)}{\partial \textbf{n}}& =& 0,& x \in \partial \Gamma .\\ \end{array} \right. \end{aligned}$$

$$\square$$

### Global attractivity of IEs

Next, we investigate whether the IEs are globally attractive using Lyapunov functions. Here, we consider the method of constructing these Lyapunov functions through the following cases:

#### ${\delta _S=0~~ and ~~\delta _I>0}$

In this case, the following Lyapunov functional is taken into consideration:

26 $$\begin{aligned} L(t)=L_1(t)+L_2(t), \end{aligned}$$

with

$$\begin{aligned} \begin{array}{l} L_1(t)=\displaystyle \int _\Gamma I^*(x)\left\{ S^*(x) h\left( \frac{S_x}{S^*(x)}\right) +I^*(x) h\left( \frac{I_x}{I^*(x)}\right) \right\} dx,\\ L_2(t)=\displaystyle \int _\Gamma I^*(x)\left\{ \varepsilon b_2 S^*(x) e^{-b_4\tau } \int _0 ^\tau h\left( \frac{S_{s,x}}{S^*(x)}\right) ds\right\} dx. \end{array} \end{aligned}$$

The *t*-derivative of $L_1(t)$ is

$$\begin{array}{lll} \frac{dL_1}{dt}& =& \displaystyle \int _\Gamma I^*(x)\left\{ \left( 1-\frac{S^*(x)}{S_x} \right) \frac{\partial S_x}{\partial t}+\left( 1-\frac{I^*(x)}{I_x} \right) \frac{\partial I_x}{\partial t}\right\} ,\\ & =& \displaystyle \int _\Gamma I^*(x)\left\{ \left( 1-\frac{S^*(x)}{S_x}\right) (b_3-b_1 S_xI_x-(b_4+b_2) S_x+\varepsilon b_2 e^{-b_4\tau }S_{\tau ,x} )\right. \\ & & \left. +\left( 1-\frac{I^*(x)}{I_x} \right) \left( \delta _{I} \displaystyle \int _\Gamma J(x-y)I_ydy+b_1 S_xI_x-(\delta _I+b_4+b_6+b_5) I_x \right) \right\} dx. \end{array}$$

Using the IEs property, which is

$$\begin{aligned} \left\{ \begin{array}{llll} b_3 = b_1 S^*(x)I^*(x)+(b_4+b_2-\epsilon b_2 e^{-b_4\tau }) S^*(x),\\ (\delta _I \tilde{J}(x) +b_4+b_5+b_6) I^*(x)=\delta _{I} \displaystyle \int _\Gamma J(x-y)I^*(y)dy+ b_1 S^*(x)I^*(x),\\ \end{array} \right. \end{aligned}$$

we get

$$\begin{array}{lll} \frac{dL_1}{dt}& =& \displaystyle \int _\Gamma I^*(x)\left\{ \left( 1-\frac{S^*(x)}{S_x}\right) (b_1 S^*(x)I^*(x)+(b_4+b_2-\epsilon b_2 e^{-b_4\tau }) S^*(x))\right. \\ & & \left. +\left( 1-\frac{S^*(x)}{S_x}\right) (-b_1 S_xI_x-(b_4+b_2) S_x+\varepsilon b_2 e^{-b_4\tau }S_{\tau ,x} )\right. \\ & & \left. +\left( 1-\frac{I^*(x)}{I_x} \right) \left( \delta _{I} \displaystyle \int _\Gamma J(x-y)I_ydy+b_1 S_xI_x-(\delta _I+b_4+b_6+b_5) I_x \right) \right\} dx,\\ & =& \displaystyle \int _\Gamma I^*(x)\left\{ (b_4+b_2)\left( 2-\frac{S^*(x)}{S_x}-\frac{S_x}{S^*(x)}\right) +b_1 S^*(x)I^*(x)\left( 1-\frac{S^*(x)}{S_x}+\frac{I_x}{I^*(x)}-\frac{ S_xI_x}{S^*(x)I^*(x)}\right) \right. \\ & & +\epsilon b_2 e^{-b_4\tau } S^*(x)\left( -1+\frac{S^*(x)}{S_x}+\frac{S_{\tau ,x}}{S^*(x)}-\frac{S_{\tau ,x}}{S_x}\right) \\ & & \left. +\left( 1-\frac{I^*(x)}{I_x} \right) \left( \delta _{I} \displaystyle \int _\Gamma J(x-y)I_ydy+b_1 S_xI_x\right) + \left( 1-\frac{I_x}{I^*(x)} \right) \left( \delta _I+b_4+b_6+b_5 \right) I^*(x) \right\} dx,\\ & =& \displaystyle \int _\Gamma I^*(x)\left\{ (b_4+b_2)\left( 2-\frac{S^*(x)}{S_x}-\frac{S_x}{S^*(x)}\right) +b_1 S^*(x)I^*(x)\left( 1-\frac{S^*(x)}{S_x}+\frac{I_x}{I^*(x)}-\frac{ S_xI_x}{S^*(x)I^*(x)}\right) \right. \\ & & +\epsilon b_2 e^{-b_4\tau } S^*(x)\left( -1+\frac{S^*(x)}{S_x}+\frac{S_{\tau ,x}}{S^*(x)}-\frac{S_{\tau ,x}}{S_x}\right) \\ & & +\left( 1-\frac{I^*(x)}{I_x} \right) \left( \delta _{I} \displaystyle \int _\Gamma J(x-y)I_ydy+b_1 S_xI_x\right) \\ & & \left. + \left( 1-\frac{I_x}{I^*(x)} \right) \left( \delta _{I} \displaystyle \int _\Gamma J(x-y)I^*(y)dy+ b_1 S^*(x)I^*(x)\right) \right\} dx,\\ & =& \displaystyle \int _\Gamma I^*(x)\left\{ (b_4+b_2+b_1 S^*(x) I^*(x))\left( 2-\frac{S^*(x)}{S_x}-\frac{S_x}{S^*(x)}\right) \right. \\ & & +\epsilon b_2 e^{-b_4\tau } S^*(x)\left( -1+\frac{S_x}{S^*(x)}+\frac{S_{\tau ,x}}{S^*(x)}-\frac{S_{\tau ,x}}{S_x}\right) \\ & & \left. +\left( 1-\frac{I^*(x)}{I_x} \right) \delta _{I} \displaystyle \int _\Gamma J(x-y)I_ydy + \left( 1-\frac{I_x}{I^*(x)} \right) \delta _{I} \displaystyle \int _\Gamma J(x-y)I^*(y)dy\right\} dx. \end{array}$$

Now, we calculate $\frac{d }{d t}\tilde{L}_2(t)$, which is given as

$$\begin{array}{lll} \frac{d }{d t}\tilde{L}_2(t)& =& \frac{d }{d t}\displaystyle \int _\Gamma \varepsilon b_2 e^{-b_4\tau }S^*(x) I^*(x) \displaystyle \int _0 ^\tau h\left( \frac{S_{s,x}}{S^*(x)}\right) dsdx,\\ & =& \displaystyle \int _\Gamma \varepsilon b_2 e^{-b_4\tau }S^*(x) I^*(x) \frac{\partial }{\partial t}\displaystyle \int _0 ^\tau h\left( \frac{S_{s,x}}{S^*(x)}\right) dsdx . \end{array}$$

Note that

$$\begin{array}{lll} \frac{\partial }{\partial t}\displaystyle \int _0 ^\tau h\left( \frac{S_{s,x}}{S^*(x)}\right) ds & =& \displaystyle \int _0 ^\tau \frac{\partial }{\partial t} h\left( \frac{S_{s,x}}{S^*(x)}\right) ds,\\ & =& -\displaystyle \int _0 ^\tau \frac{\partial }{\partial s} h\left( \frac{S_{s,x}}{S^*(x)}\right) ds,\\ & =& -\left. h\left( \frac{S_{s,x}}{S^*(x)}\right) \right| _0 ^\tau ,\\ & =& - \frac{S_{\tau ,x}}{S^*(x)}+\frac{S_x}{S^*(x)}+\ln \left( \frac{S_{\tau ,x}}{S_x}\right) . \end{array}$$

Thus, we get

$$\begin{array}{lll} \frac{d }{d t}\tilde{L}_2(t)= & \displaystyle \int _\Gamma \varepsilon b_2 e^{-b_4\tau }S^*(x) I^*(x) \left[ - \frac{S_{\tau ,x}}{S^*(x)}+\frac{S_x}{S^*(x)}+\ln \left( \frac{S_{\tau ,x}}{S_x}\right) \right] dsdx. \end{array}$$

Therefore, $$\frac{d }{d t}\tilde{L}(t)$$ becomes

$$\begin{array}{lll} \frac{d }{d t}\tilde{L}(t)& =& \displaystyle \int _\Gamma I^*(x)\left\{ (b_4+b_2+b_1 S^*(x) I^*(x))\left( 2-\frac{S^*(x)}{S_x}-\frac{S_x}{S^*(x)}\right) \right. \\ & & +\epsilon b_2 e^{-b_4\tau } S^*(x)\left( -1+\frac{S^*(x)}{S_x}+\frac{S_{\tau ,x}}{S^*(x)}-\frac{S_{\tau ,x}}{S_x}\right) \\ & & \left. +\left( 1-\frac{I^*(x)}{I_x} \right) \delta _{I} \displaystyle \int _\Gamma J(x-y)I_ydy + \left( 1-\frac{I_x}{I^*(x)} \right) \delta _{I} \displaystyle \int _\Gamma J(x-y)I^*(y)dy\right\} dx\\ & & +\displaystyle \int _\Gamma \varepsilon b_2 e^{-b_4\tau }S^*(x) I^*(x) \left\{ - \frac{S_{\tau ,x}}{S^*(x)}+\frac{S_x}{S^*(x)}+\ln \left( \frac{S_{\tau ,x}}{S_x}\right) \right\} dsdx ,\\ & =& \displaystyle \int _\Gamma I^*(x)\left\{ \left[ (b_4+b_2 )S^*(x)+b_1 S^*(x) I^*(x)-\epsilon b_2 e^{-b_4\tau } S^*(x)\right] \left( 2-\frac{S^*(x)}{S_x}-\frac{S_x}{S^*(x)}\right) \right. \\ & & -\epsilon b_2 e^{-b_4\tau } S^*(x) h\left( \frac{S_{\tau ,x}}{S_x}\right) +\left( 1-\frac{I^*(x)}{I_x} \right) \delta _{I} \displaystyle \int _\Gamma J(x-y)I_ydy \\ & & \left. + \left( 1-\frac{I_x}{I^*(x)} \right) \delta _{I} \displaystyle \int _\Gamma J(x-y)I^*(y)dy\right\} dx ,\\ & =& \displaystyle \int _\Gamma I^*(x)\left\{ b_3\left( 2-\frac{S^*(x)}{S_x}-\frac{S_x}{S^*(x)}\right) \right. -\epsilon b_2 e^{-b_4\tau } S^*(x)h\left( \frac{S_{\tau ,x}}{S_x}\right) \\ & & \left. +\left( 1-\frac{I^*(x)}{I_x} \right) \delta _{I} \displaystyle \int _\Gamma J(x-y)I_ydy + \left( 1-\frac{I_x}{I^*(x)} \right) \delta _{I} \displaystyle \int _\Gamma J(x-y)I^*(y)dy\right\} dx , \end{array}$$

Now, we put

$$\begin{aligned} M(t)= & \displaystyle \int _\Gamma I^*(x)\left\{ \left( 1-\frac{I^*(x)}{I_x} \right) \delta _{I} \displaystyle \int _\Gamma J(x-y)I_ydy + \left( 1-\frac{I_x}{I^*(x)} \right) \delta _{I} \displaystyle \int _\Gamma J(x-y)I^*(y)dy\right\} dx,\\= & \delta _{I} \displaystyle \int _\Gamma \displaystyle \int _\Gamma J(x-y) I^*(y) I^*(x)\left\{ 1-\dfrac{I_x}{I^*(x)}+\dfrac{I_y}{I^*(y)} -\dfrac{I^*(x)}{I_x}\dfrac{I_y}{I^*(y)}\right\} dx dy. \end{aligned}$$

We then obtain *M*(*t*) by changing the order of integration

$$\begin{aligned} M(t)= & \delta _{I} \displaystyle \int _\Gamma \displaystyle \int _\Gamma J(x-y) I^*(y) I^*(x)\left\{ 1-\dfrac{I_x}{I^*(x)} +\dfrac{I_y}{I^*(y)}-\dfrac{I^*(x)}{I_x} \dfrac{I_y}{I^*(y)}\right\} dx dy, \\= & \delta _{I}\displaystyle \int _\Gamma \displaystyle \int _\Gamma J(y-x) I^*(x) I^*(y) \left\{ 1-\dfrac{I_y}{I^*(y)} +\dfrac{I_x}{I^*(x)}-\dfrac{I^*(y)}{I_y} \dfrac{I_x}{I^*(x)}\right\} dx dy, \\= & \delta _{I}\displaystyle \int _\Gamma \displaystyle \int _\Gamma J(x-y)I^*(y) I^*(x) \left\{ 1-\dfrac{I_y}{I^*(y)} +\dfrac{I_x}{I^*(x)}-\dfrac{I^*(y)}{I_y} \dfrac{I_x}{I^*(x)}\right\} dx dy, \\= & \frac{1}{2}\delta _{I}\displaystyle \int _\Gamma \displaystyle \int _\Gamma J(x-y)I^*(y) I^*(x) \left\{ 1-\dfrac{I_x}{I^*(x)} +\dfrac{I_y}{I^*(y)}-\dfrac{I^*(x)}{I_x} \dfrac{I_y}{I^*(y)} \right. \\ & \left. +1-\dfrac{I_y}{I^*(y)}+\dfrac{I_x}{I^*(x)} -\dfrac{I^*(y)}{I_y}\dfrac{I_x}{I^*(x)}\right\} dx dy, \\= & \frac{1}{2}\delta _{I}\displaystyle \int _\Gamma \int _\Gamma J(x-y)I^*(y) I^*(x) \left\{ 2-\dfrac{I^*(x)}{I_x} \dfrac{I_y}{I^*(y)}-\dfrac{I^*(y)}{I_y} \dfrac{I_x}{I^*(x)}\right\} dx dy,\\\le & 0. \end{aligned}$$

Therefore, $\frac{d }{d t}\tilde{L}(t)\le 0$. Moreover, $\frac{d }{d t}\tilde{L}(t)=0$ gives $I_x=I^*(x)$ and $S_{\tau ,x}=S$,  $S_x=S^*(x)$ for $x\in \bar{\Gamma }$. Thus, we conclude that $(S^*,I^*)$ is globally attractive in $\Sigma _0$. Uniqueness is obtained directly as a result of the uniqueness of the limit.

#### ${\delta _S>0~~ and ~~\delta _I=0}$

In this case, we work with the following Lyapunov functional:

27 $$\begin{aligned} \bar{L}(t)=\bar{L}_1(t)+\bar{L}_2(t), \end{aligned}$$

with

$$\begin{aligned} \begin{array}{l} \bar{L}_1(t)=\displaystyle \int _\Gamma S^*(x)\left\{ S^*(x) h\left( \frac{S_x}{S^*(x)}\right) +I^*(x) h\left( \frac{I_x}{I^*(x)}\right) \right\} dx,\\ \bar{L}_2(t)=\displaystyle \int _\Gamma \varepsilon b_2 (S^*(x))^2 e^{-b_4\tau } \int _0 ^\tau h\left( \frac{S_{s,x}}{S^*(x)}\right) dsdx. \end{array} \end{aligned}$$

Differentiating $\bar{L}_1(t)$ by time, we get

$$\begin{array}{lll} \frac{d\bar{L}_1}{dt}& =& \displaystyle \int _\Gamma S^*(x)\left\{ \left( 1-\frac{S^*(x)}{S_x} \right) \frac{\partial S_x}{\partial t}+\left( 1-\frac{I^*(x)}{I_x} \right) \frac{\partial I_x}{\partial t}\right\} ,\\ & =& \displaystyle \int _\Gamma S^*(x)\left\{ \left( 1-\frac{S^*(x)}{S_x}\right) (\delta _S \Delta S_x+b_3-b_1 S_xI_x-(b_4+b_2) S_x\right. \\ & & \left. +\varepsilon b_2 e^{-b_4\tau }S_{\tau ,x} ) +\left( 1-\frac{I^*(x)}{I_x} \right) \left( b_1 S_xI_x-(b_4+b_6+b_5) I_x \right) \right\} dx. \end{array}$$

The positive equilibrium state verifies

$$\begin{aligned} \left\{ \begin{array}{llll} (b_4+b_2) S^*(x)=b_3 + \delta _S \Delta S^*(x)-b_1 S^*(x)I^*(x)+\epsilon b_2 e^{-b_4\tau } S^*(x),& x\in \Gamma \\ (b_4+b_5+b_6) I^*(x)= b_1 S^*(x)I^*(x),& x\in \Gamma ,\\ {\frac{\partial S^*(x)}{\partial \textbf{n}}=0,}& {x \in \partial \Gamma }. \end{array} \right. \end{aligned}$$

We get

$$\begin{array}{lll} \frac{d\bar{L}_1}{dt}& =& \displaystyle \int _\Gamma \left\{ \left( 1-\frac{S^*(x)}{S_x}\right) (\delta _S \Delta S_x+b_3+\varepsilon b_2 e^{-b_4\tau }S_{\tau ,x}) + (b_4+b_2) S^*(x)\left( 1-\frac{S_x}{S^*(x)}\right) \right. \\ & & \left. -\left( 1-\frac{S^*(x)}{S_x} \right) b_1 S_xI_x +\left( 1-\frac{I^*(x)}{I_x} \right) b_1 S_xI_x +\left( 1-\frac{I_x}{I^*(x)} \right) (b_4+b_6+b_5) I^*(x) \right\} dx,\\ & =& \displaystyle \int _\Gamma S^*(x)\left\{ \left( 1-\frac{S^*(x)}{S_x}\right) (\delta _S \Delta S_x+b_3+\varepsilon b_2 e^{-b_4\tau }S_{\tau ,x})-\left( 1-\frac{S^*(x)}{S_x} \right) b_1 S_xI_x \right. \\ & & \left. + \left[ b_3 + \delta _S \Delta S^*(x)-b_1 S^*(x)I^*(x)+\epsilon b_2 e^{-b_4\tau } S^*(x) \right] \left( 1-\frac{S_x}{S^*(x)}\right) \right. \\ & & \left. +\left( 1-\frac{I^*(x)}{I_x} \right) b_1 S_xI_x +\left( 1-\frac{I_x}{I^*(x)} \right) b_1 S^*(x)I^*(x) \right\} dx,\\ & =& \displaystyle \int _\Gamma S^*(x)\left\{ b_3\left( 2-\frac{S^*(x)}{S_x}-\frac{S_x}{S^*(x)}\right) \right. \\ & & +\epsilon b_2 e^{-b_4\tau } S^*(x)\left( 1-\frac{S_x}{S^*(x)}+\frac{S_{\tau ,x}}{S^*(x)}-\frac{S_{\tau ,x}}{S_x}\right) \left. +\delta _S \Delta S_x\left( 1-\frac{S^*(x)}{S_x}\right) +\left( 1-\frac{S_x}{S^*(x)}\right) \delta _S \Delta S^*(x)\right\} dx. \end{array}$$

Now, we calculate $\frac{d }{d t}\bar{L}_2(t)$, which is given as

$$\begin{array}{lll} \frac{d }{d t}\bar{L}_2(t)= & \displaystyle \int _\Gamma \varepsilon b_2 e^{-b_4\tau }(S^*(x))^2 \left\{ - \frac{S_{\tau ,x}}{S^*(x)}+\frac{S_x}{S^*(x)}+\ln \left( \frac{S_{\tau ,x}}{S_x}\right) \right\} dsdx. \end{array}$$

By summing $\frac{d }{d t}\bar{L}_1(t)$ and $\frac{d }{d t}\bar{L}_2(t)$ we obtain

$$\begin{array}{lll} \frac{d }{d t}\bar{L}(t)& =& \displaystyle \int _\Gamma S^*(x)\left\{ b_3\left( 2-\frac{S^*(x)}{S_x}-\frac{S_x}{S^*(x)}\right) \right. \\ & & +\epsilon b_2 e^{-b_4\tau } S^*(x)\left( 1-\frac{S_x}{S^*(x)}+\frac{S_{\tau ,x}}{S^*(x)}-\frac{S_{\tau ,x}}{S_x}\right) \left. +\delta _S \Delta S_x\left( 1-\frac{S^*(x)}{S_x}\right) +\left( 1-\frac{S_x}{S^*(x)}\right) \delta _S \Delta S^*(x)\right\} dx\\ & & +\displaystyle \int _\Gamma \varepsilon b_2 e^{-b_4\tau }(S^*(x))^2 \left\{ - \frac{S_{\tau ,x}}{S^*(x)}+\frac{S_x}{S^*(x)}+\ln \left( \frac{S_{\tau ,x}}{S_x}\right) \right\} dsdx,\\ & =& \displaystyle \int _\Gamma S^*(x)\left\{ b_3\left( 2-\frac{S^*(x)}{S_x}-\frac{S_x}{S^*(x)}\right) \right. \\ & & -\epsilon b_2 e^{-b_4\tau } S^*(x) h\left( \frac{S_x}{S^*(x)}\right) \left. +\delta _S \Delta S_x\left( 1-\frac{S^*(x)}{S_x}\right) +\left( 1-\frac{S_x}{S^*(x)}\right) \delta _S \Delta S^*(x)\right\} dx. \end{array}$$

Now, We handle the last term using Neuman boundary conditions and the Green’s first identity. Thus, we get

$$\begin{array}{lll} \frac{d }{d t}\bar{L}(t)& =& \displaystyle \int _\Gamma S^*(x)\left\{ (b_3+b_1 S^*(x) I^*(x)-\epsilon b_2 e^{-b_4\tau } S^*(x))\left( 2-\frac{S^*(x)}{S_x}-\frac{S_x}{S^*(x)}\right) \right. \\ & & \left. -\epsilon b_2 e^{-b_4\tau } S^*(x) h\left( \frac{S_x}{S^*(x)}\right) \right\} dx\\ & & +\delta _S\left\{ \displaystyle \int _\Gamma S^*(x)\left\{ \Delta S_x\left( 1-\frac{S^*(x)}{S_x}\right) +\left( 1-\frac{S_x}{S^*(x)}\right) \Delta S^*(x)\right\} dx\right\} ,\\ & =& \displaystyle \int _\Gamma S^*(x)\left\{ (b_3+b_1 S^*(x) I^*(x)-\epsilon b_2 e^{-b_4\tau } S^*(x))\left( 2-\frac{S^*(x)}{S_x}-\frac{S_x}{S^*(x)}\right) \right. \\ & & \left. -\epsilon b_2 e^{-b_4\tau } S^*(x) h\left( \frac{S_x}{S^*(x)}\right) \right\} dx\\ & & +\delta _S\left\{ \displaystyle \int _{\partial \Gamma } S^*(x)\left( 1-\frac{S^*(x)}{S_x}\right) \nabla S_x. \textbf{n} dS-\displaystyle \int _\Gamma \nabla \left( S^*(x)\left( 1-\frac{S^*(x)}{S_x} \right) \right) .\nabla S_x dx\right. \\ & & \left. +\displaystyle \int _{\partial \Gamma } S^*(x)\left( 1-\frac{S_x}{S^*(x)}\right) \nabla S^*(x). \textbf{n} dS-\displaystyle \int _\Gamma \nabla \left( S^*(x)\left( 1-\frac{S_x}{S^*(x)} \right) \right) .\nabla S^*(x)dx\right\} ,\\ & =& \displaystyle \int _\Gamma S^*(x)\left\{ (b_3+b_1 S^*(x) I^*(x)-\epsilon b_2 e^{-b_4\tau } S^*(x))\left( 2-\frac{S^*(x)}{S_x}-\frac{S_x}{S^*(x)}\right) \right. \\ & & \left. -\epsilon b_2 e^{-b_4\tau } S^*(x) h\left( \frac{S_x}{S^*(x)}\right) \right\} dx\\ & & -\delta _S\displaystyle \int _\Gamma ^n\left( \frac{d S^*(x)}{d x}-2\frac{S^*(x)}{S_x}\frac{\partial S^*}{\partial x}+\frac{(S^*(x))^2}{(S_x)^2} \frac{\partial S_x}{\partial x} \right) \frac{\partial S_x}{\partial x}dx \\ & & -\delta _S\displaystyle \int _\Gamma \left( \frac{d S^*(x)}{d x}-\frac{S_x}{S^*(x)}\right) \frac{\partial S^*(x)}{\partial x}dx,\\ & =& \displaystyle \int _\Gamma S^*(x)\left\{ (b_3+b_1 S^*(x) I^*(x)-\epsilon b_2 e^{-b_4\tau } S^*(x))\left( 2-\frac{S^*(x)}{S_x}-\frac{S_x}{S^*(x)}\right) \right. \\ & & \left. -\epsilon b_2 e^{-b_4\tau } S^*(x) h\left( \frac{S_x}{S^*(x)}\right) \right\} dx\\ & & -\delta _S\displaystyle \int _\Gamma \left( \frac{d S^*(x)}{d x}-\frac{S^*(x)}{S_x}\frac{\partial S_x}{\partial x} \right) ^2dx,\\ & \le & 0. \end{array}$$

Therefore, $\frac{d }{d t}\bar{L}(t)\le 0$. Moreover, $\frac{d }{d t}\bar{L}(t)=0$ gives $I_x=I^*(x)$ and $S_{\tau ,x}=S^*(x)$, and $S_x=S^*(x)$ for $x\in \bar{\Gamma }$. It adheres to $(S^*,I^*)$ being globally attractive in $\Sigma _0$. We obtain the uniqueness through the uniqueness of the limit directly.

#### All parameters are constants and ${\delta _S>0~~ and ~~\delta _I>0}$

In this case, we suppose that the model parameters are constant for all $x\in \bar{\Omega }$. In this case, $R_0$ becomes

$$R_0=\frac{b_1 b_3 }{(b_1+b_2-b_2 \epsilon e^{-b_4\tau })(b_4+b_5+b_6)}.$$

and the IEs satisfies

28 $$\begin{aligned} \left\{ \begin{array}{llll} (b_4+b_2-\epsilon b_2 e^{-b_4\tau }) S^*+b_1 S^*I^*=b_3,\\ (b_4+b_5+b_6) I^*= b_1 S^*I^*.\\ \end{array} \right. \end{aligned}$$

For the global attractivity of this steady state, we consider the following Lyapunov functional:

29 $$\begin{aligned} \hat{L}(t)=\hat{L}_1(t)+\hat{L}_2(t), \end{aligned}$$

with

$$\begin{aligned} \begin{array}{ll} \hat{L}_1(t)=\displaystyle \int _\Gamma \left\{ S^* h\left( \frac{S_x}{S^*}\right) +I^* h\left( \frac{I_x}{I^*}\right) \right\} dx,&\hat{L}_2(t)=\displaystyle \int _\Gamma \varepsilon b_2 (S^*)^2 e^{-b_4\tau } \int _0 ^\tau h\left( \frac{S_{s,x}}{S^*}\right) dsdx. \end{array} \end{aligned}$$

Differentiating $\hat{L}_1(t)$ with respect to *t*, we obtain

$$\begin{array}{lll} \frac{d\hat{L}_1}{dt}& =& \displaystyle \int _\Gamma \left\{ \left( 1-\frac{S^*}{S_x} \right) \frac{\partial S_x}{\partial t}+\left( 1-\frac{I^*}{I_x} \right) \frac{\partial I_x}{\partial t}\right\} ,\\ & =& \displaystyle \int _\Gamma \left\{ \left( 1-\frac{S^*}{S_x}\right) (\delta _S \Delta S_x+b_3-b_1 S_xI_x-(b_4+b_2) S_x\right. \\ & & \left. +\varepsilon b_2 e^{-b_4\tau }S_{\tau ,x} ) +\left( 1-\frac{I^*}{I_x} \right) \left( \delta _{I} \mathbb {H}[I](t,x)+b_1 S_xI_x-(b_4+b_6+b_5) I_x \right) \right\} dx. \end{array}$$

Using the equilibrium equations (28), to obtain

$$\begin{array}{lll} \frac{dL_1}{dt}& =& \displaystyle \int _\Gamma \left\{ \left( 1-\frac{S^*}{S_x}\right) (b_1 S^*I^*+(b_4+b_2-\epsilon b_2 e^{-b_4\tau }) S^*-b_1 S_xI_x-(b_4+b_2) S_x+\varepsilon b_2 e^{-b_4\tau }S_{\tau ,x} )\right. \\ & & \left. +\left( 1-\frac{I^*}{I_x} \right) \left( b_1 S_xI_x-(b_4+b_6+b_5) I_x \right) \right\} dx+ d_2\displaystyle \int _\Gamma \left( 1-\frac{I^* }{I_x} \right) \mathbb {H}[I](t,x)dx\\ & & +d_1\displaystyle \int _\Gamma \left( 1-\frac{S^* }{S_x} \right) d_2 \Delta S_xdx ,\\ & =& \displaystyle \int _\Gamma I^*(x)\left\{ (b_4+b_2+b_1 S^*(x) I^*(x))\left( 2-\frac{S^*(x)}{S_x}-\frac{S_x}{S^*(x)}\right) \right. \\ & & \left. +\epsilon b_2 e^{-b_4\tau } S^*(x)\left( -1+\frac{S_x}{S^*(x)}+\frac{S_{\tau ,x}}{S^*(x)}-\frac{S_{\tau ,x}}{S_x}\right) \right\} dx+ d_2\displaystyle \int _\Gamma \left( 1-\frac{I^* }{I_x} \right) \mathbb {H}[I](t,x)dx\\ & & -d_1\displaystyle \int _\Gamma S^* \frac{|\nabla S_x|^2}{S_x^2}dx, \end{array}$$

Next, we deal with the term $\displaystyle \int _\Omega \left( 1-\frac{I^* }{I_x} \right) \mathbb {H}[I](t,x)dx$, we obtain

$$\begin{array}{ll} \displaystyle \int _\Omega \left( 1-\frac{I^* }{I_x} \right) \mathbb {H}[I](t,x)dx& = \displaystyle \int _\Omega \displaystyle \int _\Omega J(x-y) (I_y-I_x)dy dx - I^* \displaystyle \int _\Omega \displaystyle \int _\Omega J(x-y) \frac{I_y-I_x}{I_x} dy dx,\\ \\ & =-\frac{I^*}{2} \displaystyle \int _\Omega \displaystyle \int _\Omega J(x-y) \frac{I_y-I_x}{I_x} dy dx - \frac{I^*}{2} \displaystyle \int _\Omega \displaystyle \int _\Omega J(x-y) \frac{I_y-I_x}{I_x} dy dx,\\ \\ & = -\frac{I^*}{2} \displaystyle \int _\Omega \displaystyle \int _\Omega J(x-y) \frac{I_y-I_x}{I_x} dy dx - \frac{I^*}{2} \displaystyle \int _\Omega \displaystyle \int _\Omega J(x-y) \frac{I_x-I_y}{I_y} dy dx,\\ \\ & = \frac{I^*}{2} \displaystyle \int _\Omega \displaystyle \int _\Omega J(x-y) \bigg ( 2 -\frac{I_y}{I_x}-\frac{I_x}{I_y}\bigg ) dy dx,\\ \\ & \le 0. \end{array}$$

Now, we calculate $\frac{d }{d t}\hat{L}_2(t)$, which is given as

$$\begin{array}{lll} \frac{d }{d t}\hat{L}_2(t)= & \displaystyle \int _\Gamma \varepsilon b_2 e^{-b_4\tau }(S^*(x))^2 \left\{ - \frac{S_{\tau ,x}}{S^*(x)}+\frac{S_x}{S^*(x)}+\ln \left( \frac{S_{\tau ,x}}{S_x}\right) \right\} dsdx. \end{array}$$

By summing $\frac{d }{d t}\bar{L}_1(t)$ and $\frac{d }{d t}\bar{L}_2(t)$ we obtain Therefore, $\frac{d }{d t}\tilde{L}(t)$ becomes

$$\begin{array}{lll} \frac{d }{d t}\tilde{L}(t)& =& \frac{I^*}{2} \displaystyle \int _\Omega \displaystyle \int _\Omega J(x-y) \bigg ( 2 -\frac{I_y}{I_x}-\frac{I_x}{I_y}\bigg ) dy dx -d_1\displaystyle \int _\Gamma S^* \frac{|\nabla S_x|^2}{S_x^2}dx\\ & & +\displaystyle \int _\Gamma \left\{ b_3\left( 2-\frac{S^*}{S_x}-\frac{S_x}{S^*}\right) -\epsilon b_2 e^{-b_4\tau } S^*h\left( \frac{S_{\tau ,x}}{S_x}\right) \right\} dx \end{array}$$

Therefore, $\frac{d }{d t}\hat{L}(t)\le 0$. Moreover, equality holds if and only if$I_x=I^*$ and $S_{\tau ,x}=S^*$, and $S_x=S^*$ for $x\in \bar{\Gamma }$. It adheres to $(S^*,I^*)$ being globally attractive in $\Sigma _0$.

##### Remark 3

In the foregoing analysis, we have examined two limiting cases: (i) the case of purely local diffusion, where $\delta _S > 0$ and $\delta _I = 0$, and (ii) the case of purely nonlocal diffusion, where $\delta _S = 0$ and $\delta _I > 0$. These cases were considered in order to separately assess the individual effects of each dispersal mechanism on the global dynamics of the system. Nonetheless, the general case in which both $\delta _S > 0$ and $\delta _I > 0$ holds is more biologically realistic and theoretically intricate.

It is important to emphasize that, in the local diffusion case, the Lyapunov functional is constructed using a test function of the form $S^*(x)$, which appears as the integrand weight in the functional $\bar{L}(t)$. Conversely, in the nonlocal diffusion case, the corresponding test function is $I^*(x)$, associated with the integral expression $L(t)$. In the general setting with both local and nonlocal dispersal, the principal difficulty lies in identifying an admissible test function that ensures the non-positivity of the time derivative of the Lyapunov functional.

Under the additional simplifying assumption that all model parameters are constant, the optimal test function can be chosen to be the constant function $1$ (or any other strictly positive constant), which significantly facilitates the analysis. However, in the fully general case with spatially heterogeneous coefficients, the construction of a suitable Lyapunov functional or test function remains an open and challenging problem.

## Relationship between local and corresponding nonlocal diffusion

A given nonnegative bounded function $K\in L^1(\mathbb {R}^N)$ with $N\ge 1$, and $supp K\subset \overline{B(0,1)}$, $\displaystyle \int _{\mathbb {R}^N} K(x)dx=1.$ For every $\xi >0$, we define

$$K_\xi (x):=\frac{1}{\xi ^N}K\left( \frac{x}{\xi }\right) ,~~x\in \mathbb {R}^N.$$

The function $K_\xi (x)$ is called modifiers. A function $\vartheta \in L_{loc} ^1(\Gamma )$, we define $\vartheta _\xi$ as

$$\vartheta _\xi :=(\vartheta \star K_\xi )(x)=\int _\Gamma K_\xi (x-y)\vartheta (y)dy,$$

with $y\in \Gamma _\xi := \{x\in \Gamma :dist(x,\partial \Gamma )>\xi \}.$ The function $\vartheta _\xi :\Gamma _\xi \rightarrow \mathbb {R}$ is called a mollification function of $\vartheta$.

We consider that *J*(.) takes the form $J(x)=\frac{1}{\xi }K_\xi (\frac{x}{\xi })$ with $0<\xi<<1$, $supp K \subset [-1,1]$ and $N=1$. We have the Taylor’s formula given by

$$\begin{array}{llll} \displaystyle \int _\mathbb {R} J(x-y)(I_y- I_x)dy& =& \displaystyle \int _\mathbb {R} \frac{1}{\xi }K_\xi \left( \frac{x-y}{\xi }\right) (I_y- I_x)dy,\\ & =& \displaystyle \int _\mathbb {R} K_\xi (-s)(I(t,x+\xi s)- I_x)ds,\\ & =& \frac{\partial ^2}{\partial x^2}I_x\frac{\xi ^2}{2}\displaystyle \int _\mathbb {R} K_\xi (-s)s^2ds+ \frac{\partial }{\partial x}I_x\xi \displaystyle \int _\mathbb {R} K_\xi (-s)sds+o(\xi ^2),\\ & =& D_1\Delta I_x + D_2 \frac{\partial }{\partial x}I_x+o(\xi ^2), \end{array}$$

is obtained due to the smoothness of *I*, with $\Delta =\frac{\partial ^2}{\partial x^2}$, $D_1=\frac{\xi ^2}{2}\displaystyle \int _\mathbb {R} K_\xi (-s)s^2ds$, and $D_2=\xi \displaystyle \int _\mathbb {R} K_\xi (-s)sds$. Obviously, if $D_2=0$, the nonlocal diffusion becomes the corresponding local diffusive one. Then, the nonlocal diffusion generalizes the results of the local diffusion.

## The situation of the epidemic influenced by the protection force

This section will discuss the protection force required to reduce the $\mathfrak {R}_0$ defined by (40) to a value less than one. In the case of $\mathfrak {R}_0<1$, the global stability of $E^0$ (which is the target result) is obtained. The focal point of this section is to suppose that the basic reproduction number corresponding to *SIR* epidemic model (denoted by $\mathfrak {R}_0 ^{SIR}$) is larger than one and seeking condition on the protection force $b_2$ such that $\mathfrak {R}_0$ becomes less than one, where in this case we get the global extinction of the disease. Consider the basic reproduction number corresponding to the SIR epidemic model, which is obtained by taking $b_2=0$ in (40), as follows:

30 $$\begin{aligned} \mathfrak {R}_0 ^{SIR}=\sup _{\psi \in L^2 (\Gamma )}\left\{ \frac{ \displaystyle \int _\Gamma b_1 S^0 _{SIR}(x)(\psi (x))^2dx}{\frac{1}{2}\delta _I \displaystyle \int _\Gamma \displaystyle \int _\Gamma J(x-y)(\psi (y)-\psi (x))^2dydx+\displaystyle \int _\Gamma (\delta _{I} +b_4+b_5 +b_6)(\psi (x))^2dx}\right\} , \end{aligned}$$

where the following elliptic problem:

31 $$\begin{aligned} \left\{ \begin{array}{lll} 0=\delta _S \Delta S^0 _{SIR}(x)+ b_3-b_4 S^0 _{SIR}(x),& x \in \Gamma ,\\ \frac{\partial S^0 _{SIR}(x)}{\partial \textbf{n}}=0,& x \in \partial \Gamma . \end{array}\right. \end{aligned}$$

has a solution $S^0 _{SIR}(x)$. By a simple comparison principle, we get $S^0 _{SIR}(x)\ge S^0 (x)$, $x\in \bar{\Gamma }$. Therefore, we get $\mathfrak {R}_0 ^{SIR}\ge \mathfrak {R}_0$. The corresponding elliptic problem to (30) is

32 $$\begin{aligned} \begin{array}{lll} \delta _I \mathbb {H}[\psi _1](x)+ (b_4+b_5+b_6)\psi _1(x)-\frac{b_1 S^0 _{SIR}(x)}{\mathfrak {R}_0 ^{SIR}}\psi _1(x)=0,~~x \in \Gamma , \end{array} \end{aligned}$$

where $\psi _1>0$ that corresponds to the principal eigenvalue $\mathfrak {R}_0 ^{SIR}$. Similarly, the corresponding eigenfunction to $\mathfrak {R}_0$ is $\psi _2$, which is strictly positive and verifies the following problem:

33 $$\begin{aligned} \begin{array}{lll} -\delta _I \mathbb {H}[\psi _2](x) - (b_4+b_5+b_6)\psi _2(x)+\frac{b_1 S^0(x)}{\mathfrak {R}_0 }\psi _2(x)=0,~~x \in \Gamma . \end{array} \end{aligned}$$

By multiplying (32) by $\psi _2$ and (33) by $\psi _1$ and summing them, and then integrating the obtained equation over $\Gamma$, we get

$$\frac{1}{\mathfrak {R}_0}\displaystyle \int _\Gamma b_1 S^0(x)\psi _1(x)\psi _2(x)dx =\frac{1}{\mathfrak {R}_0 ^{SIR}}\displaystyle \int _\Gamma b_1 S^0 _{SIR}(x)\psi _1(x)\psi _2(x)dx.$$

Hence,

$$\mathfrak {R}_0=\frac{\mathfrak {R}_0 ^{SIR}\displaystyle \int _\Gamma b_1 S^0(x)\psi _1(x)\psi _2(x)dx}{\displaystyle \int _\Gamma b_1 S^0 _{SIR}(x)\psi _1(x)\psi _2(x)dx},$$

which is the relationship between the $\mathfrak {R}_0$ and $\mathfrak {R}_0 ^{SIR}$. By considering $\mathfrak {R}_0<1$ (and taking into consideration $\mathfrak {R}_0 ^{SIR}>1$), we get

34 $$\begin{aligned} \displaystyle \int _\Gamma b_1 S^0(x)\psi _1(x)\psi _2(x)dx<\frac{1}{\mathfrak {R}_0 ^{SIR}}\displaystyle \int _\Gamma b_1 S^0 _{SIR}(x)\psi _1(x)\psi _2(x)dx. \end{aligned}$$

It can be observed that the right-hand side of (34) has no protection force $b_2$ (for more details, see the elliptic equation (31)). Therefore, we get $\mathfrak {R}_0<1$ whenever $b_2$ verifies the inequality (34), indicating that the protection measure controls the epidemic.

For more explanation, we consider the particular case $\delta _S=0$ and $b_2=\tilde{b_2}>0$. In this case, we obtain

$$S^0(x)=\frac{b_3}{b_4+\tilde{b_2}\left( 1-\varepsilon e^{-b_4\tau }\right) },$$

and

$$S^0 _{SIR}(x)=\frac{b_3}{b_4},$$

hence, the inequality (34) becomes $F(\tilde{b_2})<\frac{\tilde{F}}{\mathfrak {R}_0 ^{SIR}}$, with

$$F(\tilde{b_2})=\displaystyle \int _\Gamma b_1\frac{b_3}{b_4+\tilde{b_2}\left( 1-\varepsilon e^{-b_4\tau }\right) } \psi _1(x)\psi _2(x)dx,~~\tilde{F}=\displaystyle \int _\Gamma b_1\frac{b_3}{b_4}\psi _1(x)\psi _2(x)dx.$$

Notice that $\tilde{F}$ is constant with respect to $\tilde{b_2}$. Clearly, *F* is a decreasing function in $\tilde{b_2}$ and $F(0)=\tilde{F}$, and based on the fact that $\mathfrak {R}_0 ^{SIR}>1$, we have $F(0)>\frac{\tilde{F}}{\mathfrak {R}_0 ^{SIR}}$. Since $\lim _{\tilde{b_2}\rightarrow \infty }F(\tilde{b_2})=0$, we infer that $\exists$  $\tilde{b_2}_{\min }>0$ such that $F(\tilde{b_2}_{min})=\frac{\tilde{F}}{\mathfrak {R}_0 ^{SIR}}$. Moreover, for $\tilde{b_2}<\tilde{b_2}_{min}$ the inequality (34) is not verified (means $\mathfrak {R}_0>1$), and for $\tilde{b_2}>\tilde{b_2}_{min}$, the inequality (34) holds (means $\mathfrak {R}_0<1$). Therefore, we deduce that the protection can help in stopping the disease.

### Remark 4

In our model, the time delay $\tau$ represents the duration of the protection period gained by fear from infection. When $\tau$ tends to $\infty$, the term $e^{-b_4 \tau }$ tends to zero. Consequently, $S^0$ tends to $\tilde{S}^0$ as $\tau \rightarrow \infty$. By the comparison principle, we have that

$$S^0 \ge \tilde{S}^0 \text { for all } \tau > 0.$$

Substituting this estimate into the variational formulation of the basic reproduction number $R_0$, by following the aforementioned methodology, shows that the delay influences $R_0$. However, the reduction remains modest, since $0< e^{-b_4 \tau } < 1$ and its influence on the principal eigenvalue is limited.

## Numerical investigation of the results

**Fig. 1:**
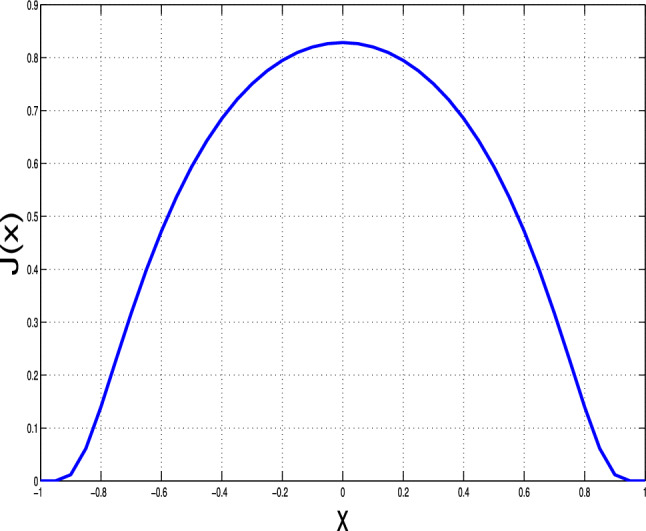
The nonlocal diffusion kernel *J*(*x*).

We let $\Gamma =(-1,1)$ and

$$J(x)={\left\{ \begin{array}{ll} Ze^{\frac{1}{x^2-1}}, \qquad & -1<x<1, \\ 0,& \text{ otherwise, } \end{array}\right. }$$

where $Z\approx 2.2523$. Note that *J* fulfills **(A4)** and its graphical representation over $\Gamma$ is shown in Fig. 1

Now, we test the theoretical results in the subsections given below.

### Stability of IFEs

This subsection is meant to check the results of section “IFEs”. Therefore, let us take the parameters into consideration that are given below:

35 $$\begin{aligned} \begin{array}{lll} b_3=2+1.5\cos (8x),~~ b_2=0.5+0.1\cos (0.78x),~~ b_5=1+0.1\cos (1.25x),~~ b_6=0.1+0.1\cos (2x),\\ b_4=0.11+0.1\cos (1.23x), ~~b_1=0.3+0.1\cos (3x), ~~ \delta _S=0.01, ~~ \delta _I=0.2,~~ \tau =10,~~ \varepsilon =0.5,\\ S(s,x)=2.5(x^2-1)\cos (x)+0.1(s+1)+3,~~ I(0,x)=2.05(x^2-1)\cos (x)+3.21. \end{array} \end{aligned}$$

**Fig. 2:**
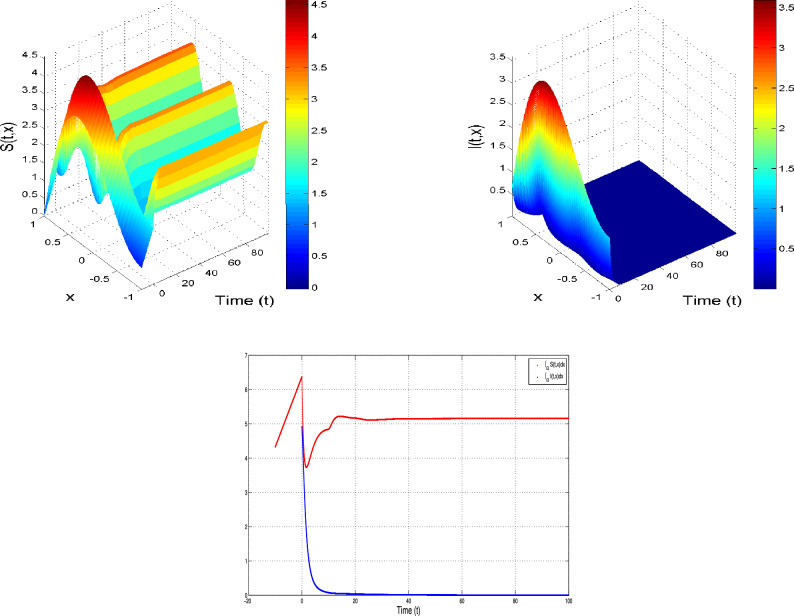
Global asymptotic stability of IFEs for the set of parameters (35). Therefore, $\mathfrak {R}_0\approx 0.783$.

Then we get $\mathfrak {R}_0\approx 0.783$, showing that $E^0$ is globally asymptotically stable, as it is depicted in Fig. 2

### Global asymptotic stability of the IEs

In Section “IEs”, the global asymptotic stability of the IEs has been shown in two cases:

- $$\mathbf {\delta _S=0~~ and ~~\delta _I>0}$$.
- $$\mathbf {\delta _S>0~~ and ~~\delta _I=0}$$.

For this aim, we consider the subsections:

#### ${\delta _S=0~~ and ~~\delta _I>0}$

We take the following set of parameters:

36 $$\begin{aligned} \begin{array}{lll} b_3=2+1.5\cos (8x),~~ b_2=0.5+0.1\cos (0.78x),~~ b_5=1+0.1\cos (1.25x),~~ b_6=0.1+0.1\cos (2x),\\ b_4=0.11+0.1\cos (1.23x), ~~b_1=1.3+0.1\cos (3x), ~~ \delta _S=0, ~~ \delta _I=0.2,~~ \tau =10,~~ \varepsilon =0.5,\\ S(s,x)=2.5(x^2-1)\cos (x)+0.1(s+1)+3,~~ I(0,x)=2.05(x^2-1)\cos (x)+3.21. \end{array} \end{aligned}$$

**Fig. 3:**
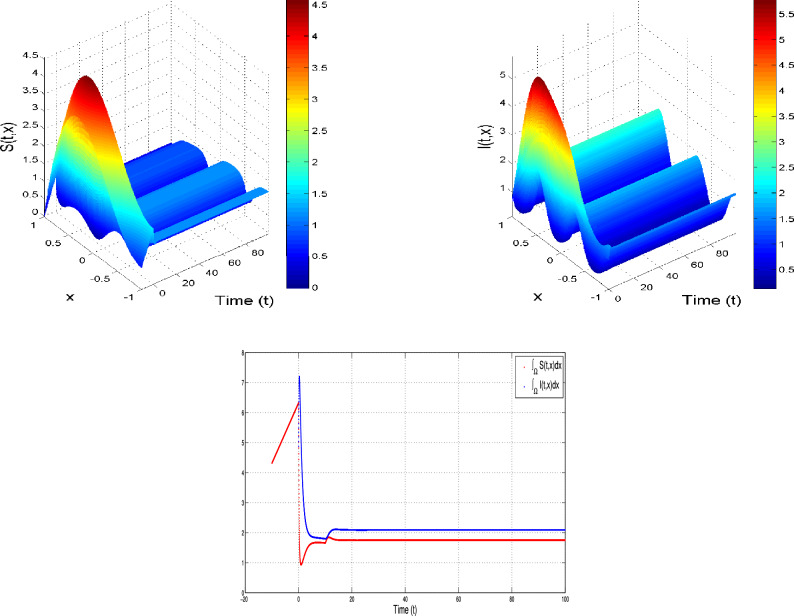
The globally asymptotically stable IEs $(S^*(x),I^*(x))$ with the set of parameters (36). Therefore, $\mathfrak {R}_0\approx 1.543$.

Thus, a IEs that is globally asymptotically stable is obtained, where $\mathfrak {R}_0\approx 1.543$ as highlighted in Fig. 3

#### ${\delta _S>0~~ and ~~\delta _I=0}$

We take similar parameters (36) and the following values:

37 $$\begin{aligned} \begin{array}{lll} \delta _S=0.2, ~~ \delta _I=0. \end{array} \end{aligned}$$

**Fig. 4:**
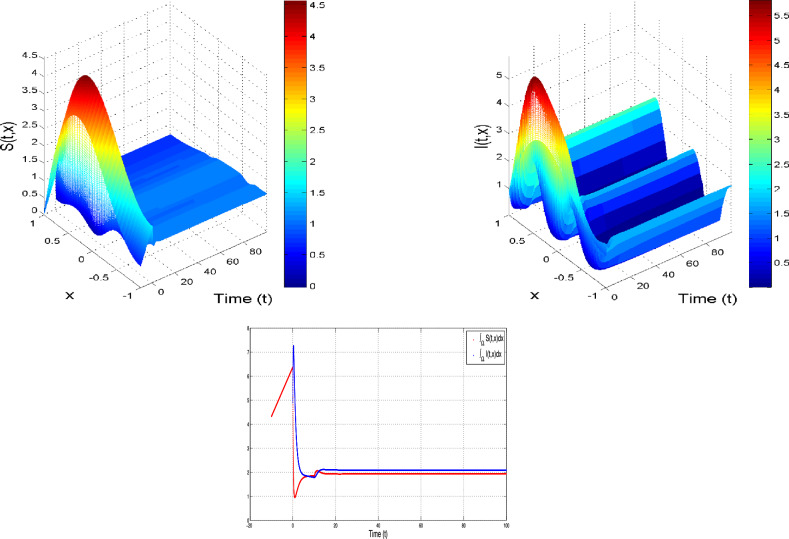
The IEs $(S^*(x),I^*(x))$ is globally stable for the set of parameters (36), where $\mathfrak {R}_0\approx 1.72$.

Thus, for the given case, a IEs is globally asymptotically stable is obtained, where $\mathfrak {R}_0\approx 1.72$ as highlighted in Fig. 4

### Required protection force to contain the epidemic

This section determines the protection force required to stop the epidemic, which is studied in section “The situation of the epidemic influenced by the protection force”. The set of parameters given below is taken into consideration:

38 $$\begin{aligned} \begin{array}{lll} b_3=2+1.5\cos (8x),~~ b_5=1+0.1\cos (1.25x),~~ b_6=0.1+0.1\cos (2x), ~~b_4=0.11+0.1\cos (1.23x),\\ b_1=1.3+0.1\cos (3x), ~~ \delta _S=0.02, ~~ \delta _I=0.2,~~ \tau =10,~~ \varepsilon =0.5,\\ S(s,x)=2.5(x^2-1)\cos (x)+0.1(s+1)+3,~~ I(0,x)=2.05(x^2-1)\cos (x)+3.21. \end{array} \end{aligned}$$

And we assume that $b_2$ takes the form

$$b_2(x)=\alpha _0(1+0.1\cos (0.78x)).$$

**Fig. 5:**
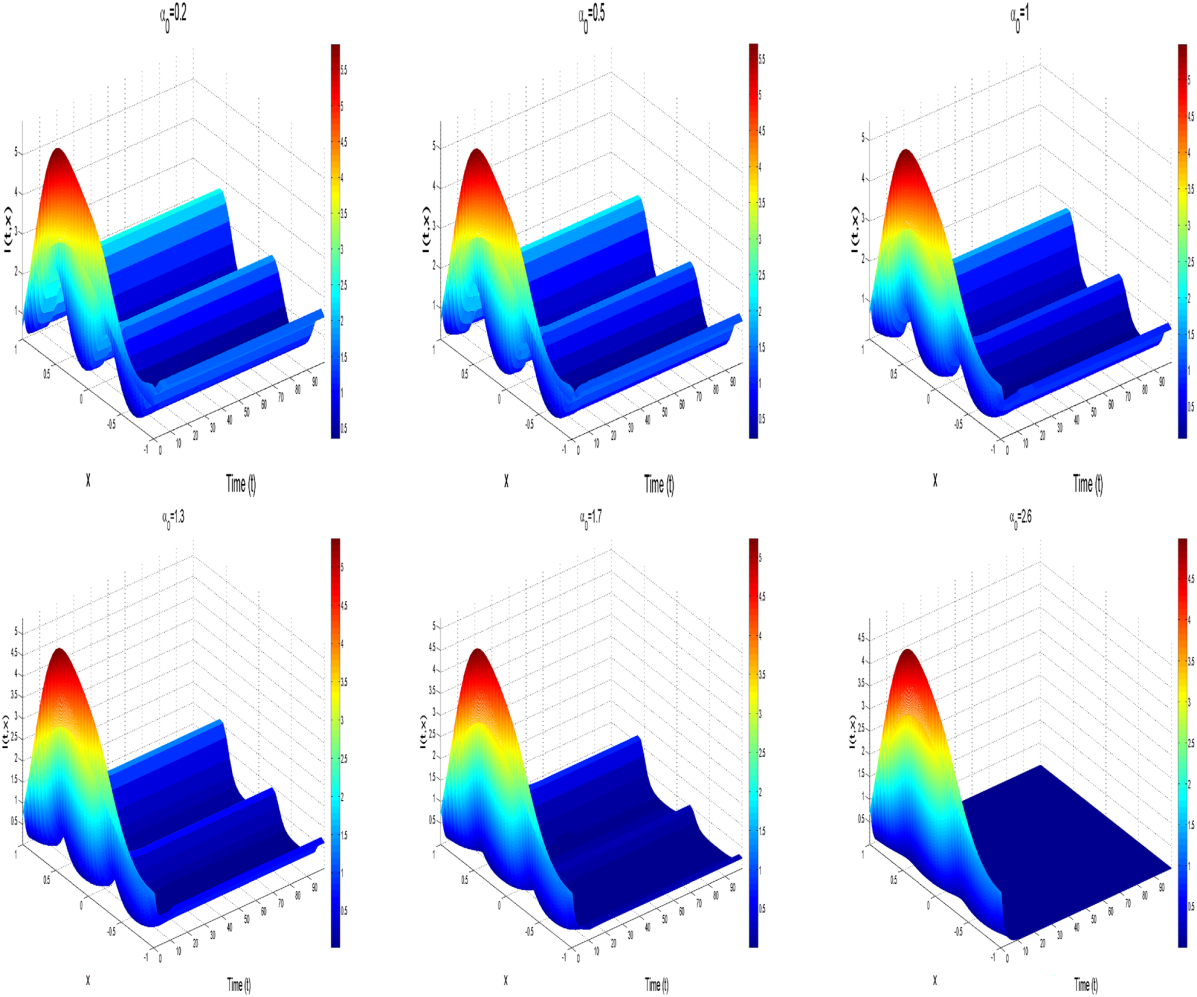
The effect of protection function $b_2$ on stopping the epidemic for the set of parameters (39), where for $\alpha _0=0.2$, we get $\mathfrak {R}_0\approx 1.78$ and for $\alpha _0=2.6$, we get $\mathfrak {R}_0\approx 0.89$. Clearly, the situation forwards from the epidemic for $\alpha _0=0.2$ to the extinction of disease for $\alpha _0=2.6$.

Indeed, for $\alpha _0=0$, we get the results obtained by the classical *SIR* epidemic model. We hope that by augmenting the value of $\alpha _0$, we will reach the desired protection force required to stop the epidemic. That means the inequality (34) holds, which means that the IFEs become globally asymptotically stable. For these regards, we plot Fig. 5.

### Effect of the kernel *J* on the basic reproduction number

Motivated by the work in^27^, we consider the following examples of the kernel function $J$, all of which satisfy assumption **(A4)**:

$$\begin{aligned} \text {(1) Gaussian kernel:} \quad&J(x) = \frac{1}{\sqrt{2\pi \sigma ^2}} e^{-\frac{x^2}{2\sigma ^2}}, \\ \text {(2) Laplace (exponential) kernel:} \quad&J(x) = \frac{1}{2\lambda } e^{-\frac{|x|}{\lambda }}, \\ \text {(3) Epanechnikov kernel:} \quad&J(x) = {\left\{ \begin{array}{ll} \frac{3}{4}(1 - x^2), & \text {if } |x| \le 1, \\ 0, & \text {otherwise}, \end{array}\right. } \\ \text {(4) Triangular kernel:} \quad&J(x) = {\left\{ \begin{array}{ll} 1 - |x|, & \text {if } |x| \le 1, \\ 0, & \text {otherwise}, \end{array}\right. } \\ \text {(5) Raised cosine kernel:} \quad&J(x) = {\left\{ \begin{array}{ll} \frac{\pi }{4} \cos \left( \frac{\pi x}{2}\right) , & \text {if } |x| \le 1, \\ 0, & \text {otherwise}. \end{array}\right. } \end{aligned}$$

**Fig. 6:**
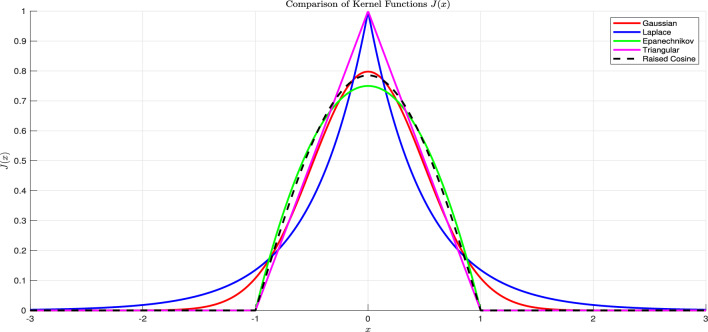
Comparison of different types of the kernel function $J$.

These kernel functions are depicted graphically in Fig. 6.

We consider the following set of parameters:

39 $$\begin{aligned} \begin{array}{lll} b_3 = 0.41, \quad b_4 = 0.11, \quad b_5 = 0.1, \quad b_6 = 0.1, \quad \delta _S = 0.02, \quad \delta _I = 5.5, \\ \tau = 10, \quad \epsilon = 0.5, \quad b_2 = 0.5. \end{array} \end{aligned}$$

Moreover, we assume that $b_1$ takes the spatially heterogeneous form

$$b_1(x) = \alpha _0 \left( 1 + 0.1 \cos (0.78x)\right) ,$$

and the corresponding equilibrium value $S^0$ is given by

$$S^0 = \frac{b_3}{b_4 + b_2 - \epsilon e^{-b_4 \tau }}.$$

Accordingly, the variational formulation of the basic reproduction number reads

40 $$\begin{aligned} \mathfrak {R}_0 = \sup _{\psi \in L^2 (\Gamma )}\left\{ \frac{ b_1(x) S^0 \displaystyle \int _\Gamma \psi (x)^2 \, dx}{ \delta _I \displaystyle \int _\Gamma \int _\Gamma J(x-y)\left( \psi (y)-\psi (x)\right) ^2 dy dx + (b_4 + b_5 + b_6) \displaystyle \int _\Gamma \psi (x)^2 \, dx} \right\} . \end{aligned}$$

By evaluating the expression (40) numerically for each of the five kernels, we obtain the following values for $\mathfrak {R}_0$:

- Gaussian kernel: $$\mathfrak {R}_0 = 2.2520$$,
- Laplace kernel: $$\mathfrak {R}_0 = 1.9085$$,
- Epanechnikov kernel: $$\mathfrak {R}_0 = 2.3571$$,
- Triangular kernel: $$\mathfrak {R}_0 = 2.4500$$,
- Raised cosine kernel: $\mathfrak {R}_0 = 2.3853$.

These results highlight the significant impact of the kernel function $J$ on the value of the basic reproduction number, and thus on the underlying dynamics of the epidemic model.

## Conclusion

The fear of the spread of infection always resides globally because of its effective transmission from the infected population to the susceptible ones. Although a small number of individuals are prone to get infected as fear grows, we need to find some methodologies that can reduce the spread of any infectious disease by minimizing the transmission of infection. In this paper, we have introduced a novel SPIR epidemic model that couples mathematical modeling of the infectious disease and the impact of its induced fear on the social interactions and behavior changes in the population by providing protection as a safety measure against all odds. Moreover, it contains both local and nonlocal diffusions and investigates the effect of fear on the mobility of the population by taking $\delta _1=0$ and $\delta _2=0$, one at a time.

We proved that this SPIR model is well-posed and has a globally defined solution. The existence of a global compact attractor in $\mathbb {X}_+$ is strongly supported by the fact that the semiflow given by $\{\Theta (t)\}_{t\ge 0}$ is $\varkappa -$condensing map. For the threshold quantity $\mathfrak {R}_0$, we have obtained the global extinction of the disease, i.e., the IFEs are globally asymptotically stable for $\mathfrak {R}_0 <1$. The global persistence of the disease, i.e., the SPIR model under consideration, is uniformly persistent for $\mathfrak {R}_0 >1$ and has an IE that is $E^*$.

We have further found the required minimal protection rate $b_{2min}$ which is needed to stop the epidemic by reducing the $\mathfrak {R}_0$ below one in accordance with the assumption $\mathfrak {R}^{SIR}_0 >1$. In the last part of the paper, we illustrated our findings by numerical simulation that shows that the disease eventually dies out by protecting a precise portion of the healthy population.

We strongly urge that the study in this paper can help investigate the spread, transmission, fear rate, and the minimum protection needed for contagious infections from a very small scale to a large scale, such as COVID-19 disease. Although protection may vary from one disease to another, it is still needed to stop the disease. The most commonly used measures include isolation, vaccination, or confinement, which can affect the findings under consideration.

## Data Availability

The datasets used and/or analysed during the current study available from the corresponding author on reasonable request.
